# Identification of Clusters in a Population With Obesity Using Machine Learning: Secondary Analysis of The Maastricht Study

**DOI:** 10.2196/64479

**Published:** 2025-02-05

**Authors:** Maik JM Beuken, Melanie Kleynen, Susy Braun, Kees Van Berkel, Carla van der Kallen, Annemarie Koster, Hans Bosma, Tos TJM Berendschot, Alfons JHM Houben, Nicole Dukers-Muijrers, Joop P van den Bergh, Abraham A Kroon, Maastricht Study Management, Iris M Kanera

**Affiliations:** 1 Faculty of Financial Management Research Center for Statistics & Data Science Zuyd University of Applied Sciences Sittard Netherlands; 2 Faculty of Health, School of Physiotherapy Research Center for Nutrition, Lifestyle and Exercise Zuyd University of Applied Sciences Heerlen Netherlands; 3 Department of Data Collection Research and Innovation Statistics Netherlands Heerlen Netherlands; 4 Department of Internal Medicine Maastricht University Medical Center+ Maastricht Netherlands; 5 Cardiovascular Research Institute Maastricht Maastricht University Maastricht Netherlands; 6 Department of Social Medicine Maastricht University Maastricht Netherlands; 7 Care and Public Health Research Institute Maastricht University Maastricht Netherlands; 8 University Eye Clinic Maastricht Maastricht University Maastricht Netherlands; 9 Department of Health Promotion Care and Public Health Research Institute Maastricht University Maastricht Netherlands; 10 Department of Sexual Health, Infectious Diseases and Environmental Health Living Lab Public Health Mosa Public Health Service South Limburg Heerlen Netherlands; 11 Department of Internal Medicine VieCuri Medical Center Venlo Netherlands; 12 See Acknowledgments

**Keywords:** Maastricht Study, participant clusters, cluster analysis, factor probabilistic distance clustering, FPDC algorithm, statistically equivalent signature, SES feature selection, unsupervised machine learning, obesity, hypothesis free, risk factor, physical inactivity, poor nutrition, physical activity, chronic disease, type 2 diabetes, diabetes, heart disease, long-term behavior change

## Abstract

**Background:**

Modern lifestyle risk factors, like physical inactivity and poor nutrition, contribute to rising rates of obesity and chronic diseases like type 2 diabetes and heart disease. Particularly personalized interventions have been shown to be effective for long-term behavior change. Machine learning can be used to uncover insights without predefined hypotheses, revealing complex relationships and distinct population clusters. New data-driven approaches, such as the factor probabilistic distance clustering algorithm, provide opportunities to identify potentially meaningful clusters within large and complex datasets.

**Objective:**

This study aimed to identify potential clusters and relevant variables among individuals with obesity using a data-driven and hypothesis-free machine learning approach.

**Methods:**

We used cross-sectional data from individuals with abdominal obesity from The Maastricht Study. Data (2971 variables) included demographics, lifestyle, biomedical aspects, advanced phenotyping, and social factors (cohort 2010). The factor probabilistic distance clustering algorithm was applied in order to detect clusters within this high-dimensional data. To identify a subset of distinct, minimally redundant, predictive variables, we used the statistically equivalent signature algorithm. To describe the clusters, we applied measures of central tendency and variability, and we assessed the distinctiveness of the clusters through the emerged variables using the *F* test for continuous variables and the chi-square test for categorical variables at a confidence level of α=.001

**Results:**

We identified 3 distinct clusters (including 4128/9188, 44.93% of all data points) among individuals with obesity (n=4128). The most significant continuous variable for distinguishing cluster 1 (n=1458) from clusters 2 and 3 combined (n=2670) was the lower energy intake (mean 1684, SD 393 kcal/day vs mean 2358, SD 635 kcal/day; *P*<.001). The most significant categorical variable was occupation (*P*<.001). A significantly higher proportion (1236/1458, 84.77%) in cluster 1 did not work compared to clusters 2 and 3 combined (1486/2670, 55.66%; *P*<.001). For cluster 2 (n=1521), the most significant continuous variable was a higher energy intake (mean 2755, SD 506.2 kcal/day vs mean 1749, SD 375 kcal/day; *P*<.001). The most significant categorical variable was sex (*P*<.001). A significantly higher proportion (997/1521, 65.55%) in cluster 2 were male compared to the other 2 clusters (885/2607, 33.95%; *P*<.001). For cluster 3 (n=1149), the most significant continuous variable was overall higher cognitive functioning (mean 0.2349, SD 0.5702 vs mean –0.3088, SD 0.7212; *P*<.001), and educational level was the most significant categorical variable (*P*<.001). A significantly higher proportion (475/1149, 41.34%) in cluster 3 received higher vocational or university education in comparison to clusters 1 and 2 combined (729/2979, 24.47%; *P*<.001).

**Conclusions:**

This study demonstrates that a hypothesis-free and fully data-driven approach can be used to identify distinguishable participant clusters in large and complex datasets and find relevant variables that differ within populations with obesity.

## Introduction

Overwhelming evidence shows that modern unhealthy lifestyle behaviors (eg, physical inactivity, poor nutrition, tobacco consumption) in many parts of the world increase the prevalence and incidence of obesity and chronic illnesses such as type 2 diabetes mellitus (T2DM), coronary heart disease, and some forms of cancer [1]. Prevention of (secondary) diseases and health promotion have been proposed as important solutions to ensure a sustainable health care system for the future [2,3]. Dutch national programs aiming at promoting a healthy lifestyle in a large number of people have, however, not yet led to a decrease in the prevalence of the abovementioned diseases or a decrease in health care consumption [4].

Obesity needs to be considered a complex system problem, influenced by a combination of genetic, biological, behavioral, social, economic, and environmental factors. These factors are intertwined and can reinforce each other. An unhealthy diet can be influenced by, for example, personal factors, cultural habits, the availability of healthy food, and economic conditions. To better understand the multifaceted nature of obesity, a systems approach is needed [5,6].

Research indicates that personalized interventions appear to be more effective than general programs in achieving long-term lifestyle behavior change among various populations [7-9]. Personalized treatment takes the variability among patients into account, considering, for example, genetic, environmental, disease-related, behavioral, and lifestyle-related factors to optimize treatment outcomes. In recent years, growing experience has been gained in applying advanced data analytics and machine learning (ML) in the context of public health [10]. Processing large amounts of data by including ML enables the recognition of patterns and the identification of variables that might play an important role in personalizing programs and interventions, due to the possibility to identify persons in a certain cluster or possibilities in exploring mechanisms underlying the distinctiveness between the different clusters. Moreover, emerging hypotheses may lead to additional opportunities. Recently, data-driven approaches are increasingly being explored on large datasets. ML algorithms can detect patterns in large and complex datasets, which would be difficult to find by more simple, conventional analyses. Clustering algorithms can organize similar data points into groups, uncovering patterns that may not be obvious with traditional statistical methods. Conventional statistical models, which require a priori information, often struggle with these complex systems; this restriction requires thorough data analysis and clear modeling, which can be challenging to manage in large datasets [11]. Interestingly, this kind of exploratory data analysis can be conducted without a predefined hypothesis. With regard to obesity, hypothesis-driven methods may be less suitable for understanding the complexity of this problem because they focus on isolated variables, might miss interactions, overlook broader contexts, and may not capture the multifaceted influences on obesity. Such a hypothesis-free approach provides the chance for exploration and discovery of patterns and relationships in the data without being limited or biased by a priori–defined expectations. In general, emerging patterns may be meaningful in describing complex phenomena; discovering cross-links between different variables; and identifying possible, distinct clusters within a population. Due to recent progress in processing large amounts of intricate and unorganized data, contemporary ML techniques are becoming ever more essential in the realm of personalized medicine [12].

Recently, various data clustering approaches have been applied among different populations. Nagamine et al [13] used a hypothesis-free approach to find patterns within the symptom range mentioned by patients with heart failure (n=25,861) and characterized these clusters in terms of their distinguishing variables in order to generate characteristics and progression patterns of heart failure. Nicolet et al [14] identified clusters for clinical practice to investigate the patient multimorbidity and complexity of Swiss residents aged ≥50 years (n=18,732) in claims data. Elbattah and Molloy [15] clustered older adult participants in Ireland after a hip fracture into subgroups, in order to better predict the most beneficial care strategy for each patient. Takeshita et al [16] identified 7 clusters of obesity (BMI ≥35 kg/m^2^) in order to tailor interventions based on the data in a health care claims database (n=9494). The abovementioned studies show that using unsupervised ML techniques offers unique opportunities to uncover unexpected insights, identify novel patterns, and elucidate previously unknown connections within large data by taking the approach of exploratory, data-driven, and hypothesis-free analysis. All these studies use the k-means clustering technique. Given these promising results and developments in the field of unsupervised ML techniques, we chose to include new developments in this study.

In particular, we explore in this paper the clustering of a large multidimensional dataset provided by The Maastricht Study. The Maastricht Study is an observational, prospective, population-based, cohort study [17]. Contrary to the aforementioned studies, which rely mainly on health care data, The Maastricht Study data include a broader range of potential variables, including etiology, pathophysiology, complications, and comorbidities of T2DM and other chronic diseases, and are featured by a comprehensive phenotyping approach. This dataset is complex due to the high number of variables, which implies high mathematical dimensionality, including noisy data and outliers. To handle these mathematical characteristics of the data, we choose to apply factor probabilistic distance clustering (FPDC) in this study [18].

The main aim of this study is a methodological exploration to identify potential clusters and relevant variables among individuals with obesity among participants in The Maastricht Study, using a data-driven and hypothesis-free ML approach.

## Methods

### Ethical Considerations

We used cross-sectional data from The Maastricht Study, an observational, prospective, population-based, cohort study. The rationale and methodology were described previously by Schram et al [19]. The Maastricht Study was approved by the institutional medical ethical committee (NL31329.068.10) and the Minister of Health, Welfare, and Sports of the Netherlands (Permit 131088-105234-PG). All participants gave written informed consent.

### Study Population

Eligible for inclusion into this study were participants of The Maastricht Study with obesity. Within The Maastricht Study context, individuals were enlisted through widespread media outreach efforts and by sending invitations through postal mail to those registered in municipal records as well as the regional Diabetes Patient Registry. Recruitment was structured based on the known status of T2DM, with a deliberate emphasis on selecting more individuals with T2DM to ensure operational efficiency as part of The Maastricht Study.

### Measurements

The Maastricht Study dataset includes a wide range of variables disaggregated in various general and disease-specific measurements comprising, for example, questionnaire data, physical examinations, and blood and urine examinations. For a detailed description of all measurements, see Schram et al [19].

In this study, data were included from all measured variables (2971 variables) of the cross-sectional data from the first 9188 participants who were included in the baseline survey between November 2010 and October 2020. For the specific purpose of our study, data from individuals with obesity were selected for our data analysis within this dataset based on the waist circumference variable. Men with a waist circumference higher than 102 cm (about 3.35 ft) and women with a waist circumference higher than 88 cm (about 2.89 ft) can be defined as being abdominal obese [20,21]. For a detailed description of the variables included, we refer to The Maastricht Study Dictionary [20]. This dictionary provides an overview of the variables included in our study.

### Statistical Analysis

#### Overview

**Figure 1:**
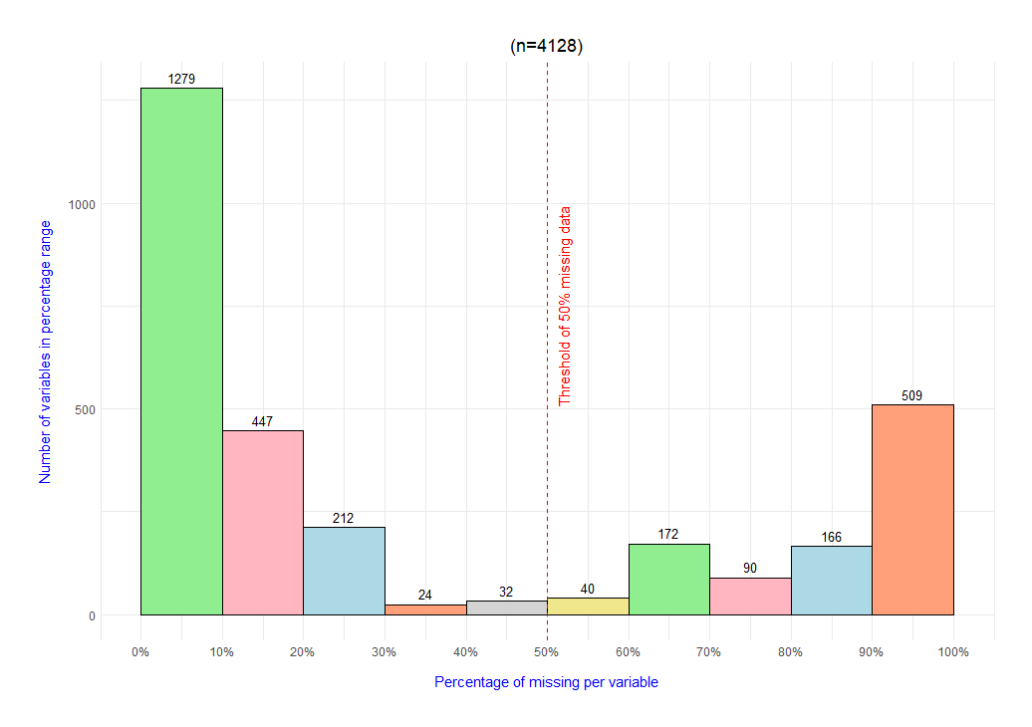
Histogram of the amount of missing data.

All data were assessed for aberrant measurement data and missing data. One of the steps to prepare the data before clustering was to remove variables with more than 50% missing data from the dataset. To provide more detail on the amount of missing data, an overview of the missingness is given in Figure 1. To impute the missing data in the remaining variables, we used the chained random forests (CRF) method [22,23] from the *missRanger* package in R. This package allows the use of predictive mean matching. Predictive mean matching ensures an imputed value that (1) occurs in the variables of interest in the case of categorical values and (2) attempts to bring the variance to a realistic size. In this research, all remaining variables were used to fill in the missing values in the data. Random forests have properties that allow them to impute heterogeneous data, unlike most standard methods, and can handle complex, nonlinear relationships in the data automatically [24], with high imputation accuracy [22]. The strength of the ensemble approach, by using random forest, lies in combining the predictions of multiple trees, which ensures a more reliable usage of the patterns underneath the data. Linear or distance-based methods struggle to capture this data structure and these underlying patterns. This feature of CRF makes the clustering, based on the decomposition into factors, more reflective of the actual data structure and underlying patterns. Another important feature of CRF is the ability to handle high-dimensional data, without overfitting [25]. This is in contrast to methods like k-nearest neighbors, which suffer in high dimensional space [26], and work well when no assumptions are made regarding the different types of missingness [27]. These properties led to the conviction that CRF is a suitable choice to impute this high-dimensional dataset when keeping the aim—to cluster the data—in mind. Further handling of missing data including data imputation is described in Multimedia Appendix 1. Data were standardized before inclusion.

#### Software

R Studio (version 4.3.0 {2023-04-21}; R Foundation for Statistical Computing) was used as an integrated development environment for the R programming language. R has been used including the packages in Textbox 1.

**List of R packages used in this study**

- *missRanger*: An approach using random forests in sequence to fill in missing values within datasets containing a mix of different data types [22,28].
- *FPDclustering*: Factor probabilistic distance clustering is a factor clustering method that involves linear transformation of variables and clusters that optimize the probabilistic distance clustering criterion [18,29,30].
- *MXM*: Many feature selection methods for a wide range of response variables, including minimal, statistically equivalent, and equally predictive feature subsets [31,32].
- *uwot*: The uniform manifold approximation and projection method for dimensionality reduction [33].
- *vtable*: Variable table for variable documentation [34].

#### Identification of Participant Clusters

A novel unsupervised ML technique developed to handle high-dimensional and complex data was used to identify clusters in data from people with obesity. We applied the FPDC algorithm with the *FPDclustering* package in R. The FPDC algorithm was used to cluster individual cases into *k* clusters after preparing the data as described in Multimedia Appendices 2 and 3. The parameter *k* is required to be established within the algorithm framework. Subsequently, clustering was conducted across a range from 2 to 30 clusters, in line with previous research [13,35]. Followed by the identification of the optimal number of clusters through evaluation criteria, the number of clusters was determined by choosing an adequate number for the practice, but also by aiming for the highest possible average silhouette coefficient (SC) score. In addition, the uniform manifold approximation and projection (UMAP) plot provides insight into the actual distinctiveness of the clustering through visualization.

This algorithm executes a linear transformation of the original variables to reduce the number of variables into orthogonal factors, the so-called Tucker-3 decomposition [36]. Thereafter, these orthogonal factors are clustered with probabilistic distance (PD) clustering [18,30]. PD clustering assigns units to a cluster according to their probability of membership. Different connections between probabilities and distances can be postulated, leading to diverse methods of clustering the data. In this study, the assumption holds that the product of the probability and the distance from each point to any cluster’s center is a constant; thus, it is more probable that a data point is assigned to a cluster when this point is closer to the cluster’s center [37,38]. The probabilistic nature of FPDC and its ability to handle transformed data (ie, Tucker-3 decomposition) allow it to be more robust to outliers [18]. Unlike some traditional deterministic methods, FPDC does not rigidly assign outliers to clusters, which can help maintain the integrity of the main clusters. This robustness is particularly useful in real-world applications where data can be noisy or contain anomalies. To overcome unstable results due to a large number of variables and correlated variables, which is a problem with PD clustering, the transformation into orthogonal factors is important. This combination results in a clustering algorithm suitable for high-dimensional datasets [39]. Besides being suited for high-dimensional datasets with correlating variables, the FPDC algorithm is also considered in this study because of the advantages it offers: (1) being a distribution-free clustering method. It outperforms models relying on k-means clustering (eg, factorial k-means) when dealing with non–Gaussian-shaped clusters or when (2) dealing with noisy data. This is due to the probabilistic approach that is more flexible and robust in handling noise in the data. Next to these advantages, the FPDC algorithm is (3) robust to outliers [18], as mentioned before. We believe that in this study the FPDC algorithm is suitable to handle outliers and noisy data, which is an important feature because of the complexity of the data used. Next to this, the FPDC algorithm is also considered because of the vast amount of variables and the amount of correlating variables, which is also a property of this dataset.

#### Evaluation of the Clustering Algorithm

To assess the outcome of the FPDC algorithm (ie, definition and number of clusters), we used the average SC, which provides a measure of both the cohesion and segregation of each data point [40]. This SC is derived from the probability matrix generated by the FPDC algorithm, which assigns a probability to each point in a cluster. To achieve this, a density-based SC is used [41]. In addition, apart from computing the SC, we depicted the data in 2 dimensions using UMAP. As the SC value approaches 1, the cohesion among data points within one cluster strengthens, while the segregation between data points within that cluster, relative to those in other clusters, increases. In conjunction with this metric, UMAP offers visualization of the data in 2D space, aiding in assessing the effectiveness of the clusters and their separation.

#### Description of Participant Clusters

In an attempt to take a data-driven and hypothesis-free approach to find and characterize the patterns in the large and complex The Maastricht Study dataset (ie, clusters), a feature selection technique was used to find the most distinguishing variables for each cluster separately. To understand each cluster’s unique combination of variables separately, after the FPDC algorithm assigned participants to clusters, we applied a feature selection model (the statistically equivalent signature [SES] algorithm [42]) for cluster *C_j_* with fixed j {1,…,*k*} based on binary logistic regression as a conditional independence test [32].

The cluster assignment is decoded in such a way that



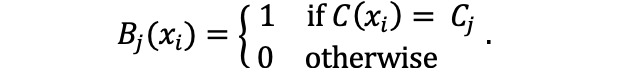



With this, a binary classification problem was thus created. The data used in this feature selection model consists of *N_j_* data points in *C_j_* and randomly selected data points from cluster subsets other than *C_j_*. To ensure that no class imbalance arises, we drew a simple random sample from the data points in the other clusters, where the ratio of the number of data points from the clusters other than cluster *C_j_* was maintained and the sum of the sampled data points equaled the number of data points in *C_j_*. Refer to Multimedia Appendix 4 for a deeper explanation. By studying the variable importance with the SES algorithm, we can make statements about which variables have a greater impact on the classification performance (ie, which variables can better distinguish *C_j_* from the other clusters). Since we used sampling, we sampled the data points 8 times and thus used the SES algorithm 8 times for each cluster separately to select the variables. We sampled 8 times so that (1) we could capture the loss of information as much as possible and (2) the randomness in selecting variables could be counteracted by being sampled.

#### Variable Selection

The SES algorithm attempts to identify predictive variables grouped into subsets, which are called signatures of the target variable, while avoiding a high degree of redundancy; it aims for a minimum size of these variable subsets and to maximize the predictive power of the variable subsets over the target variable, that is, trying to predict the binary outcome variable introduced in the previous section. The algorithm identifies multiple predictive variable subsets whose performance, in terms of predictive power, is statistically equivalent [32]. It does this by using conditional independence tests (α=.01) to assess the relationship between variables and the target variable (ie, being in a certain cluster or not) [43]. By applying these statistical tests, with an iterative forward-backward filtering technique on every variable, it will add variables that are significantly related to the target variable, conditional on already selected variables, to the subset of variables (ie, the signature) in the forward phase. It will also remove variables, after selecting a new variable, that have become redundant in their information in the subset by testing whether these variables are still significantly associated with the target variable given all other selected variables. Initially, each variable is selected in its own group. If variables seem to be interchangeable in predicting the target variable, then they will be combined into one group. The SES algorithm will pick one variable from each group to form this so-called signature. By following these steps, the SES algorithm aims to provide a minimal subset of variables that contains all the information needed to predict the target variable without adding variables that do not provide unique information to the subset of variables, considering the variables already in the subset [32,43].

In this algorithm, the use of a conditional independence test, suitable for heterogeneous data and a binary target variable, makes it possible to extend the SES algorithm to meet the specific requirements of the dataset used in this study. Therefore, we used a conditional independence test, provided in the algorithm, that is characterized by the binary target variable and the heterogeneous predictor variables it can handle, by using the binary logistic regression model to predict the target variable. Variables emerged by the SES algorithm, in terms of differences between the clusters, can be considered as a set of variables that are selected in such a way that the variables are minimally redundant in their predictive information within the subset of variables and that, as a set of variables, have maximum predictive power toward the binary classification of the outcome variable. This property is useful in terms of the aim of this study because it will return a subset of variables that is strongly related to the cluster number without providing redundant information in the subset of variables. When using a conventional statistical test for comparison between the variables in each cluster (eg, the Mann-Whitney *U* test) on all variables, it is difficult to select variables that are very distinguishable under the condition that we do not only report on variables that provide similar information. This would lead to an unsuitable interpretation of the clusters in an attempt to find cross-links between a variety of variables within the clusters. These considerations led to the use of the SES algorithm, which was introduced by Tsamardinos et al [42], on the data used in this study.

#### Description of Results

We used descriptive statistics to describe the variables of the identified clusters (mean, 5-number summary, SD, and the number and percentages) to indicate whether there is distinctiveness between participants, whether they belonging to a certain cluster or not, and the outcomes in a variable of interest. We used the group differences *F* test for continuous variables and the chi-square test of independence for categorical variables at a confidence level of α=.001. To compute these measures, we used the *sumtable* function from the *vtable* package (version 1.4.4) [34]. To ensure that it is easy to compare statistics, we created separate tables for continuous and categorical variables, resulting in 6 tables. These tables are presented in Multimedia Appendices 5-10. Although all *P* values are denoted as <.001, these values are actually all less than or equal to 1.13 x 10^-9^. All these variables were extracted from The Maastricht Study dataset; their explanations are available on the web [20].

## Results

### Overview of the study population

In total, we included 4128 eligible participants from The Maastricht Study who were obese in our data analysis (Table 1). In total, 1586 variables were included after the data preparation and thus used in the FPDC algorithm.

**Table 1:** General description of the sample (n=4128)

Variable and category		Value
**Sex, n (%)**		
	Female	2246 (54.41)
**Age (years), mean (SD)**		60.92 (8.39)
**Marital status, n (%)**		
	Divorced	345 (8.36)
	Living together	202 (4.89)
	Married	3031 (73.43)
	Other marital status	22 (0.53)
	Single (not necessarily never married)	308 (7.46)
	Widowed	220 (5.33)
**Educational level, n (%)**		
	Higher professional education or university education	1335 (32.34)
	Intermediate vocational education, higher secondary education, or higher vocational education	1878 (45.49)
	No education or primary education or lower vocational education.	915 (22.17)
**Employment status, n (%)**		
	Employed	1672 (40.5)
	Unemployed	2389 (57.87)
	Other	67 (1.62)
**Do or did you have a paid job? n (%)**		
	No	285 (6.9)
	Yes	3843 (93.1)
**Occupational category, n (%)**		
	High occupational class	496 (12.02)
	Intermediate occupational class	208 (5.04)
	Low occupational class	247 (5.98)
	Not working	2722 (65.94)
	Self-employed	446 (10.8)
	Other	9 (0.22)
**Waist circumference (cm), mean (SD)**		
	Female	99.65 (9.69)
	Male	112 (8.52)
**Diabetes status, n (%)**		
	No diabetes	1868 (45.25)
	Type 2 diabetes	1434 (34.74)
	Prediabetes	804 (19.48)
	Other types of diabetes	22 (0.53)
**Assigned numbering cluster, n (%)**		
	Cluster 1	1458 (35.32)
	Cluster 2	1521 (36.85)
	Cluster 3	1149 (27.83)

### Identification of Participant Clusters

**Figure 2:**
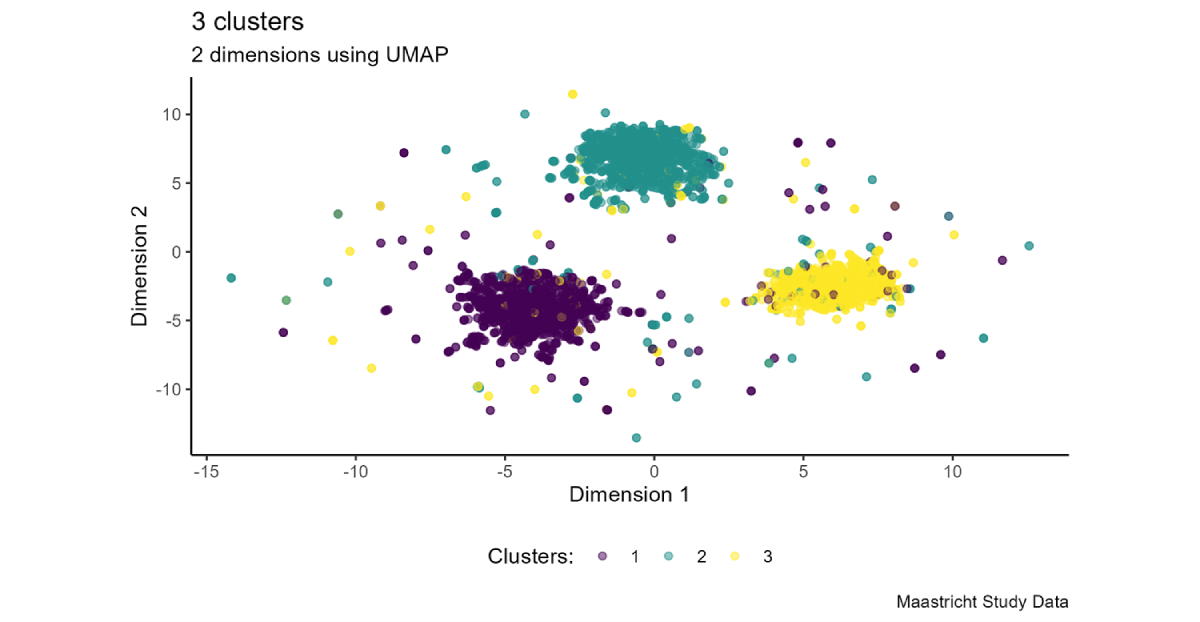
Uniform manifold approximation and projection (UMAP) on 2D of the 3 clusters.

The SC in Multimedia Appendix 11 shows that 2 and 3 clusters were identified as the most optimal clustering number by the FPDC algorithm. Figure 2 shows the results of the FPDC algorithm by a UMAP projection that displays 3 distinct clusters, each with different characteristics.

A comprehensive overview of all selected variables and how the clusters differ from the other two clusters is displayed in Multimedia Appendices 5-10. Pictured next here, a description is given of the 2 variables of each cluster with the highest *F* statistic, for continuous variables, and the highest chi-square statistic, for categorial variables, by which a cluster differed from the other 2 clusters. For more information about these variables, we refer to the online dictionary of The Maastricht Study [20].

**Figure 3:**
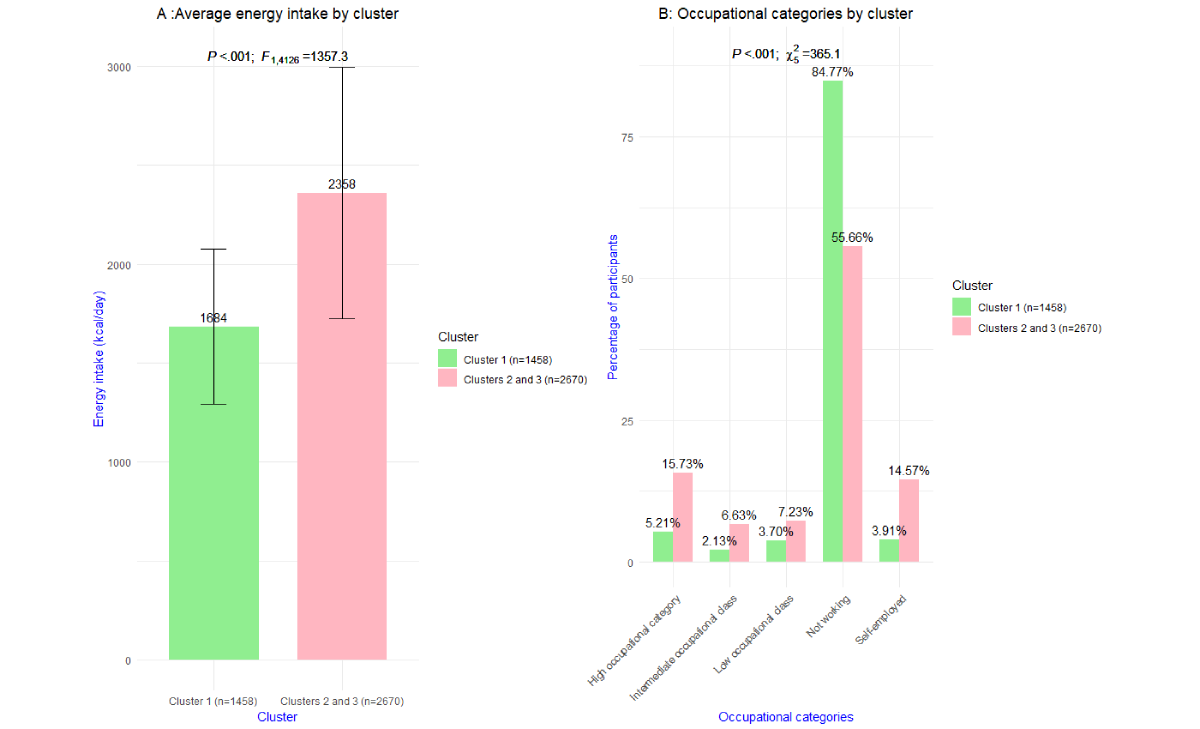
Visualization of the most significant (A) continuous and (B) categorical variable of cluster 1 versus clusters 2 and 3 combined.

Cluster 1 (1458/4128, 35.32%) reported on average a significantly lower energy intake (mean 1684, SD 393 kcal/day; *P*<.001) than the average kilocalorie intake for clusters 2 and 3 combined (mean 2358, SD 635 kcal/day). The most significant (*P*<.001) categorical variable was occupation. A larger proportion of cluster 1 (1236/1458, 84.77%) did not work compared with clusters 2 and 3 (1486/2670, 55.66%); in cluster 1, the proportion of high occupational category (76/1458, 5.21%) was smaller than that in clusters 2 and 3 (420/2670, 15.73%). A similar picture can be seen for the proportion of intermediate occupational class; of the 1458 participants in cluster 1, a total of 31 (2.13%) participants report being in an intermediate occupational class, against 177 (6.63%) of 2670 participants in clusters 2 and 3. Of all participants in cluster 1, 54 (3.7%) out of 1458 reported a low occupational class, against 193 (7.23%) of 2670 participants in cluster 2 and 3. Of 1458 participants in cluster 1, a total of 57 (3.91%) were self-employed; in the other 2 clusters, 389 (14.57%) out of 2670 participants were self-employed. In Figure 3, these results are visualized.

**Figure 4:**
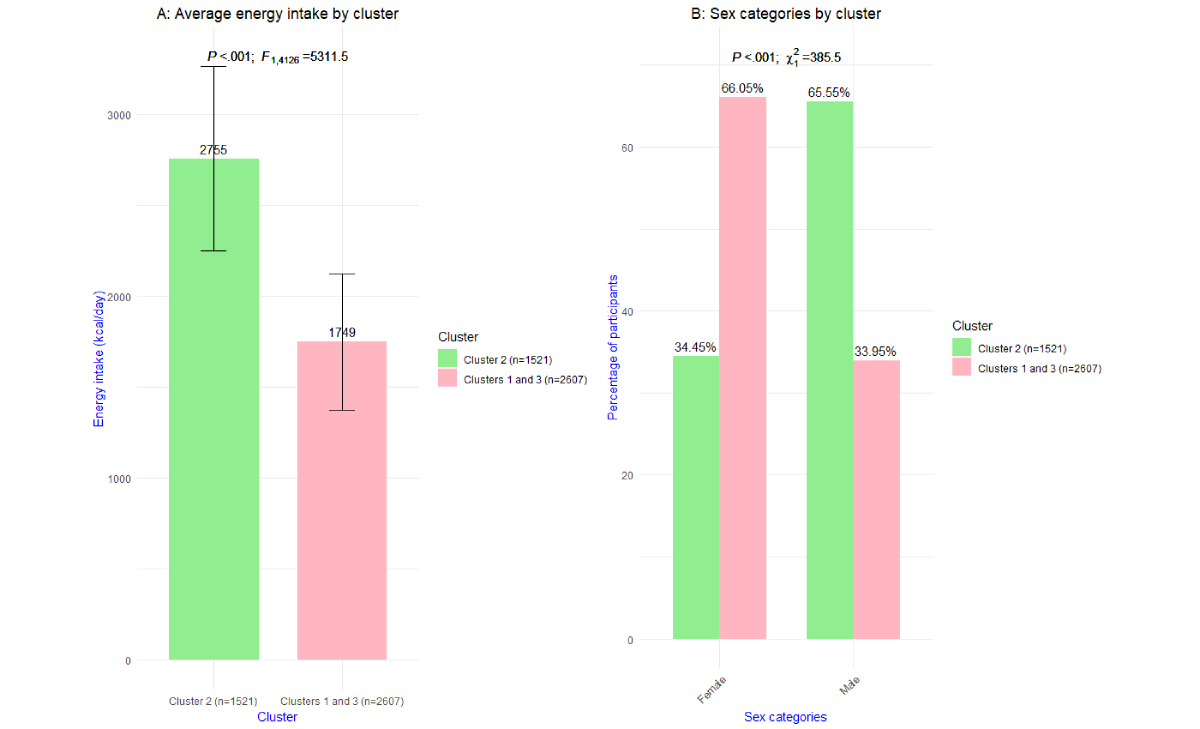
Visualization of the most significant (A) continuous and (B) categorical variable of cluster 2 versus clusters 1 and 3 combined.

Cluster 2 (1521/4128, 36.85%) included predominantly male participants. Sex was in this cluster the most significant (*P*<.001) categorical variable. A total of 997 (65.55%) of all 1521 participants in cluster 2 were male; this was in contrast to clusters 1 and 3, where 885 (33.95%) of 2607 participants were male. Looking at the most significant continuous variable, cluster 2 reported a significantly higher energy intake (mean 2755, SD 506.2 kcal/day; *P*<.001) than clusters 1 and 3 (mean 1749, SD 375 kcal/day). In Figure 4, these results are visualized.

**Figure 5:**
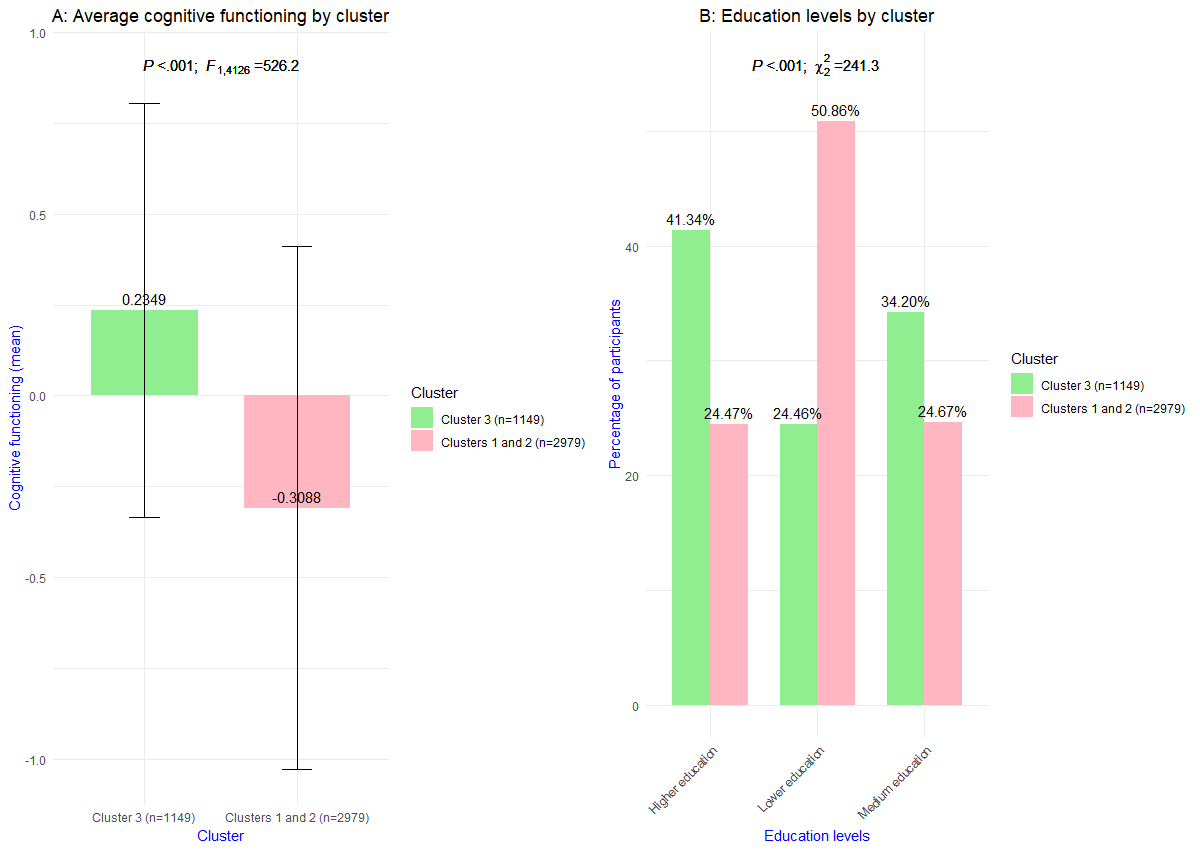
Visualization of the most significant (A) continuous and (B) categorical variable of cluster 3 versus clusters 1 and 2 combined.

Cluster 3 (1149/4128, 27.83%) reported significantly higher overall cognitive functioning (mean 0.2349, SD 0.5702; *P*<.001)) than clusters 1 and 2 combined (mean –0.3088, SD 0.7212). Educational level was the most significant categorical variable (*P*<.001). A higher proportion of cluster 3 participants received higher vocational or university education (475/1149, 41.34%) compared with the other 2 clusters combined (729/2979, 24.47%). A substantially lower proportion of cluster 3 attended no education, did not complete primary education, completed only primary education, or had lower vocational education. Of 1149 participants in cluster 3, a total of 281 (24.46%) reported this low educational level. In cluster 1 and 2 combined, 1515 (50.86%) out of 2979 participants reported the same educational level. Participants in cluster 3 received, relatively more often, intermediate vocational education or higher secondary education (393/1149, 34.2%) than the other 2 clusters combined (735/2979, 24.67%). In Figure 5, these results are visualized.

## Discussion

### Principal Findings

This study aimed to identify distinct clusters and relevant variables on which the clusters differ within a large population of individuals with obesity (n=4128) by applying an unsupervised ML algorithm. We included all available and eligible personal-level variables (2971 variables) from a population-based rich dataset of The Maastricht Study and used a hypothesis-free approach to group the data into clusters, analyzing the variables for their clustered association with obesity, without any previous human selection-based on established hypotheses.

Our novel data analysis strategy, which included (1) the FPDC algorithm, (2) SES feature selection, and (3) statistical testing, appeared valuable and successful for identifying distinct clusters within a population. This conclusion is based on the highly significant *F* and chi-square statistics observed for the selected variables across the clusters (refer to Multimedia Appendices 5-10). In this study, we chose to describe 3 clusters. This choice was based on the results of the SC and the UMAP. In this analysis strategy, we see particular strength in the use of an algorithm, specifically designed to cluster high dimensional data, in combination with the selection of a subset of minimally redundant predictive variables; this subset does not include variables that do not provide unique information in predicting the cluster numbers and has maximum predictive power over the cluster numbers generated by the clustering algorithm. This approach offers the opportunity to find relationships between the most proximate variables for obesity included in the dataset (eg, nutrition and physical activity) and the more distal variables (eg, occupation and education) that come into play in the background. This provides the opportunity to not only address the more proximal factors but also to learn about the importance of the more distal variables within the 3 different clusters. In each cluster, the root of the obesity problem might be a different one.

It should be noted, however, that the extent to which these 3 clusters could be relevant to clinical practice was not investigated in this study. To provide a meaningful interpretation and translate the findings to practical advice and programs, expert researchers, health care professionals, and representatives of those with obesity must be involved as well in field testing.

In comparison with the study of Takeshita et al [16], who also used unsupervised ML, we can point out some differences. The differences in results (eg, 7 clusters) within a population with morbid obesity may be explained by differences in the study population, type of data, and statistical analysis approach. In our study, we included individuals with obesity based on waist circumference, while Takeshita et al [16] included individuals with class 2 obesity (BMI≥35 kg/m^2^). We chose not to use BMI as a measure of obesity because BMI does not take into account fat distribution throughout the body. Waist circumference is a measure of visceral fat and is strongly associated with all-cause morbidity and mortality [44]. Even though both studies used unsupervised ML, there are differences in the statistical approach. We used the SES algorithm to identify the subset of variables that has the strongest predictive power toward the cluster number, resulting in a set of substantial distinguishing variables that are minimally redundant in their predictive information. Despite using the SES algorithm, we report variables that provide similar information (eg, energy intake reported as kilocalorie intake and kilojoule intake). This is due to using this algorithm 8 times and thus creating 8 subsets of variables for each cluster. This approach ensured the clusters were interpreted such that cross-links could be made between a variety of distinct variables within the clusters. Takeshita et al [16], on the other hand, performed a chi-square test to identify clinical variables. Nevertheless, some of our findings seem to be in line with Takeshita et al [16]. In particular, the findings of Takeshita et al [16] indicate that ocular diseases are a significant factor in differentiating cluster 4 from the other 6 obesity subgroups. In our study, we found that ocular measurements were contributing factors in the difference between cluster 1 and the other 2 clusters. In the study of Takeshita et al [16], age and sex are likely among several factors that contribute to the differences observed between the clusters. We see a similar picture in our clustering output. In Takeshita et al [16], cluster 6 is identified with the lowest hemoglobin A_1c_ (HbA_1c_) level among the clusters. In our study, we found that cluster 3 has a significantly lower HbA_1c_ level in comparison with the other 2 clusters. This suggests that HbA_1c_ is a critical factor in differentiating these clusters from others in both studies. These findings indicate a certain similarity in the cluster characteristics. However, because The Maastricht Study offers more broadly varied data, compared to Takeshita et al [16], we can report on other potential differentiating factors such as dietary intake and energy consumption, and we also can offer insights into factors like cognitive functioning, aggression, and socioeconomic status.

This study primarily centered on the data analysis strategy. In future research, the content of the clusters should be interpreted by experts in the field of obesity and the clinical relevance should be assessed.

### Limitations

Even though we used a hypothesis-free approach, data preparation was needed to apply the FPDC algorithm. For instance, The Maastricht Study data collected from participants lacked clear differentiation across all variables due to indistinguishable answer options in some variables. Essentially, these variables had significant overlap, rendering them ineffective for distinguishing between clusters. For example, the variable for ethnicity is divided into 2 categories (Caucasian or other); however, since almost 99% of the participants with obesity reported to be Caucasian, this variable is not useful to cluster on. The use of the FPDC algorithm is subject to some limitations. This algorithm can be computationally intensive, which leads to long processing times on high-dimensional data. Furthermore, this algorithm is sensitive to the choice of the initial parameters used, for example, the choice of the number of factors. In our study, we applied hyperparameter tuning to ensure a valid choice of these parameters. In addition, due to its flexibility with outliers, the FPDC algorithm may have difficulty detecting clustering structures if there is a significant size difference between clusters and the 2 clusters are not far apart. For example, if one cluster has a much larger number of units than another cluster and the clusters are not far apart, the FPDC algorithm may fail to identify the smaller cluster. Given the absence of significant differences in the number of data points across clusters in this study, we anticipate that this issue does not adversely affect our results. In our dataset, individuals diagnosed with T2D were overrepresented. In the Dutch general population, the prevalence with T2D is about 7%, but in this study, the prevalence is almost 35%. We chose not to correct for oversampling of patients with T2D in this study, because we have come to the belief that (1) this study is methodological in nature, (2) the loss of information cannot be compensated for, and (3) the objective of this study does not involve the generalization to T2D patients.

### Conclusions

This study illustrates that using a hypothesis-free approach using the FPDC algorithm successfully identified 3 distinct clusters within a complex and extensive dataset concerning a population with obesity. The SES algorithm proved to be adept at uncovering highly discriminative variables that highlight differences between the clusters identified by the FPDC algorithm. Further research and collaboration with clinical experts are needed to interpret the content of the clusters and to assess potential clinical relevance.

## Appendix

Multimedia Appendix 1 Handling missing data including imputation.

Multimedia Appendix 2 Preparing data for clustering.

Multimedia Appendix 3 Flowchart: dataset preparation.

Multimedia Appendix 4 Sampling data for the use of the SES algorithm. SES: statistically equivalent signature.

Multimedia Appendix 5 Table with cluster 1 (n=1458) compared to clusters 2 and 3 combined (n=2670), continuous variables.

Multimedia Appendix 6 Table with cluster 1 (n=1458) compared to clusters 2 and 3 combined (n=2670), categorical variables.

Multimedia Appendix 7 Table with cluster 2 (n=1521) compared to clusters 1 and 3 combined (n=2607), continuous variables.

Multimedia Appendix 8 Table with cluster 2 (n=1521) compared to clusters 1 and 3 combined (n=2607), categorical variables.

Multimedia Appendix 9 Table with cluster 3 (n=1149) against clusters 1 and 2 combined (n=2979), continuous variables.

Multimedia Appendix 10 Table with cluster 3 (n=1149) against clusters 1 and 2 combined (n=2979), categorical variables.

Multimedia Appendix 11 Silhouette coefficient per number of clusters.

## Abbreviations

CRF: chained random forests
FPDC: factor probabilistic distance clustering
HbA1c: hemoglobin A1c
ML: machine learning
PD: probabilistic distance
SC: silhouette coefficient
SES: statistically equivalent signature
T2DM: type 2 diabetes mellitus
UMAP: uniform manifold approximation and projection

## Data Availability

The dataset analyzed in this study is not publicly available due to the availability of these data, which were used under license for this study but are available from the corresponding author on reasonable request and with permission of the Maastricht Study Management Team.
