# Supplementary material for: Identification of Clusters in a Population With Obesity Using Machine Learning: Secondary Analysis of The Maastricht Study
Source: JMIR Med Inform. 2025 Feb 5;13:e64479. doi: 10.2196/64479 (PMC11840370; doi:10.2196/64479)
Supplement: Multimedia Appendix 1 [file medinform_v13i1e64479_app1.doc]

**Appendix 1.** Handling missing data including imputation

**Handling missing data**

In a first step to evaluate and analyze relevant variables in the clustering process irrelevant variables, in the sense of meta-data, are removed from the dataset. After this step and because of the precondition that complete data sets are important for performing the cluster analysis, non-responders and variables with more than 50% missing data (non-available’s; NA’s) were removed.

Since there is a certain level of complexity in this dataset because of the large number of variables and the heterogeneous character, an imputation technique based on the Random Forest prediction algorithm is used. This algorithm can handle any type of data and makes as few assumptions as possible about structural aspects of the data, which makes it suitable for high dimensional data [22]. Another important aspect of this algorithm is that it outperforms other methods for imputation when combinations of different types of missingness occur [27].
