# Supplementary material for: Identification of Clusters in a Population With Obesity Using Machine Learning: Secondary Analysis of The Maastricht Study
Source: JMIR Med Inform. 2025 Feb 5;13:e64479. doi: 10.2196/64479 (PMC11840370; doi:10.2196/64479)
Supplement: Multimedia Appendix 2 [file medinform_v13i1e64479_app2.doc]

**Appendix 2.** Preparing data for clustering

**Standardization**

To downsize effects of large size variables (or having a great variability) on cluster analysis, several standardization methods were conducted [45]. Continuous variables were standardized with a z-score-standardization method. Nominal or ordinal variables were standardized using dummy variables.

**Removing near zero variance variables**

Variables with a near zero variance were removed because they do not contribute information and therefore the minority of the values that are represented in a near zero variable could have an undue influence on the model [46]. Near-zero variance variables pertain to variables within a dataset characterized by either singular, invariant values (resulting in zero variance) or exhibiting a paucity of unique values relative to the total number of observations, in this study we used a 10% cutoff. Such variables also demonstrate a pronounced imbalance in frequency distribution, where the occurrence of the most prevalent value significantly outweighs that of the second most prevalent value. In this study we used a 95:5 cutoff for this ratio, which is the default setting in the function that is used.
