# Supplementary figures and images for: Identification of Clusters in a Population With Obesity Using Machine Learning: Secondary Analysis of The Maastricht Study
Source: JMIR Med Inform. 2025 Feb 5;13:e64479. doi: 10.2196/64479 (PMC11840370; doi:10.2196/64479)

**Appendix 3.** Flowchart: Dataset preparation


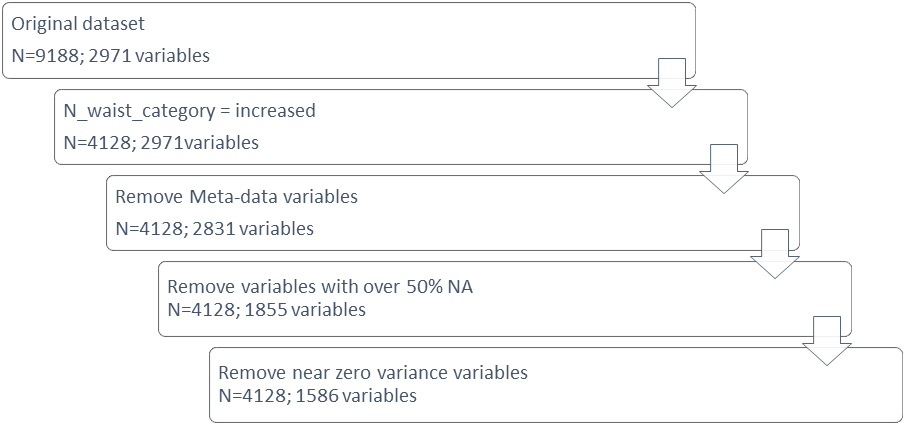

Supplement: Multimedia Appendix 3 [file medinform_v13i1e64479_app3.doc]

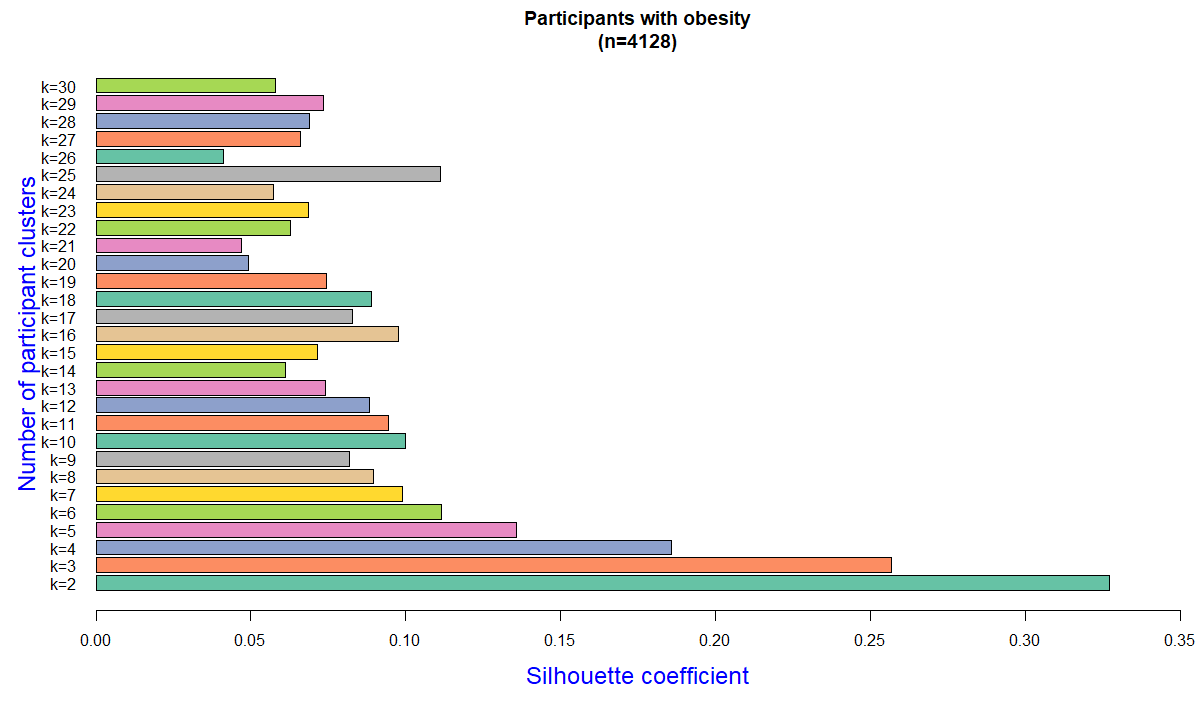

Supplement: Multimedia Appendix 11 [file medinform_v13i1e64479_app11.png]
