# Supplementary material for: Identification of Clusters in a Population With Obesity Using Machine Learning: Secondary Analysis of The Maastricht Study
Source: JMIR Med Inform. 2025 Feb 5;13:e64479. doi: 10.2196/64479 (PMC11840370; doi:10.2196/64479)
Supplement: Multimedia Appendix 4 [file medinform_v13i1e64479_app4.doc]

#### Appendix 4. Sampling data for the use of the SES algorithm

Suppose the clusters
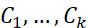
 are a partition of target population
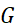
, meaning that the clusters are not empty, their union equals
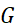
, and they are pairwise disjoint. Let
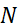
 denote the size of
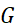
 and
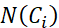
 the size of cluster
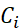
 for
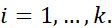
 Then


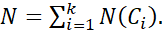


If cluster
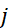
 is compared with the remaining clusters, a stratified sample with proportional allocation is drawn from the remaining clusters such that the total sample size in the remaining clusters equals the size of cluster
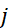
. So, denoting
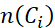
 the sample size in cluster
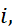


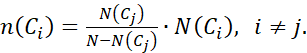


Since these numbers need not be integers, they are rounded to the nearest integers. The samples within the strata were drawn by simple random sampling without replacement. This way, a balanced group with
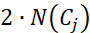
 elements is used in the SES algorithm.
