# Supplementary material for: Identification of Clusters in a Population With Obesity Using Machine Learning: Secondary Analysis of The Maastricht Study
Source: JMIR Med Inform. 2025 Feb 5;13:e64479. doi: 10.2196/64479 (PMC11840370; doi:10.2196/64479)
Supplement: Multimedia Appendix 5 [file medinform_v13i1e64479_app5.doc]

**Appendix 5.** Table with Cluster 1 (n=1458) compared to Clusters 2 and 3 combined (n=2670), continuous variables.

| **Variable** | Cluster number | Mean (SD) | Minimum | Median (IQR) | Maximum | *F* test (*df*) | *P-*value | Runs^a^ |
| --- | --- | --- | --- | --- | --- | --- | --- | --- |
|  |  |  |  |  |  |  |  |  |
| **Gait speed 6MWT (m/s)** |  |  |  |  |  |  |  |  |
|  | Cluster 1 | 1.41 (0.2361) | 0.2222 | 1.425 (1.289-1.556) | 2.111 | 542.8 (1, 4126) | <.001 | 3 |
|  | Other clusters | 1.582 (0.2214) | 0.2 | 1.592 (1.461-1.725) | 2.475 |  |  |  |
| **Mean diastolic blood pressure during 24h BP measurement** |  |  |  |  |  |  |  |  |
|  | Cluster 1 | 72.52 (7.019) | 51.94 | 72.25 (67.84-77.06) | 99.4 | 291.8 (1, 4126) | <.001 | 2 |
|  | Other clusters | 76.66 (7.671) | 53.72 | 76.18 (71.49-81.18) | 110.5 |  |  |  |
| **Coefficient of variance of systolic blood pressure during wake time (09.00-21.00h)** |  |  |  |  |  |  |  |  |
|  | Cluster 1 | 10.95 (3.769) | 4.055 | 10.21 (8.385-12.79) | 34.4 | 78.9 (1, 4126) | <.001 | 1 |
|  | Other clusters | 9.937 (3.324) | 3.798 | 9.247 (7.823-11.18) | 34.4 |  |  |  |
| **Weighted coefficient of variance of systolic blood pressure during 24h** |  |  |  |  |  |  |  |  |
|  | Cluster 1 | 10.39 (3.007) | 4.658 | 9.82 (8.28-11.97) | 27.93 | 87.0 (1, 4126) | <.001 | 1 |
|  | Other clusters | 9.536 (2.717) | 4.504 | 9.012 (7.749-10.7) | 26.15 |  |  |  |
| **Mean 7days diastolic blood pressure, evening (6.00-11.00h)** |  |  |  |  |  |  |  |  |
|  | Cluster 1 | 74.23 (8.124) | 46 | 74 (69-79) | 122 | 208.2 (1, 4126) | <.001 | 6 |
|  | Other clusters | 78.25 (8.801) | 55 | 78 (72-84) | 128 |  |  |  |
| **Cleaned Total Score Groninger Intelligence Test (GIT)^b^** |  |  |  |  |  |  |  |  |
|  | Cluster 1 | 12.46 (3.445) | 0 | 13 (10-15) | 20 | 127.3 (1, 4126) | <.001 | 2 |
|  | Other clusters | 13.62 (2.98) | 0 | 14 (12-16) | 20 |  |  |  |
| **Concept Shifting Test (CST) 2 (s)^c^** |  |  |  |  |  |  |  |  |
|  | Cluster 1 | 5.494 (1.49) | 3 | 5 (4-6) | 13 | 316.0 (1, 4126) | <.001 | 1 |
|  | Other clusters | 4.722 (1.238) | 2 | 5 (4-5) | 13 |  |  |  |
| **Concept Shifting Test (CST) total time for null version 2 (s)^c^** |  |  |  |  |  |  |  |  |
|  | Cluster 1 | 5.962 (1.435) | 3.22 | 5.715 (4.962-6.65) | 13.1 | 324.5 (1, 4126) | <.001 | 7 |
|  | Other clusters | 5.202 (1.213) | 2.44 | 4.99 (4.41-5.742) | 13.81 |  |  |  |
| **Energy intake–(kcal/day)** |  |  |  |  |  |  |  |  |
|  | Cluster 1 | 1684 (393) | 615.5 | 1684 (1410-1949) | 2937 | 1357.3 (1, 4126) | <.001 | 8 |
|  | Other clusters | 2358 (635) | 608 | 2301 (1894-2731) | 4178 |  |  |  |
| **MUFA (g/day)** |  |  |  |  |  |  |  |  |
|  | Cluster 1 | 21.08 (6.843) | 4.258 | 20.49 (16.06-25.66) | 46.23 | 1198.4 (1, 4126) | <.001 | 1 |
|  | Other clusters | 33.12 (12.29) | 5.916 | 31.5 (24.16-39.64) | 88.23 |  |  |  |
| **C18:1 cis total (mg/day)** |  |  |  |  |  |  |  |  |
|  | Cluster 1 | 17502 (5725) | 3393 | 17063 (13337-21320) | 40470 | 1177.7 (1, 4126) | <.001 | 3 |
|  | Other clusters | 27610 (10421) | 5458 | 26126 (20114-32965) | 74571 |  |  |  |
| **C18:2 conjugated total (mg/day)** |  |  |  |  |  |  |  |  |
|  | Cluster 1 | 56.2 (37.43) | 4.247 | 46.88 (32.35-67.22) | 289.5 | 372.1 (1, 4126) | <.001 | 3 |
|  | Other clusters | 95.73 (73.18) | 7.836 | 73.99 (50.86-114.4) | 709.1 |  |  |  |
| **Carbohydrates total (g/day)** |  |  |  |  |  |  |  |  |
|  | Cluster 1 | 186.4 (53.27) | 54.03 | 184 (148.6-219.4) | 408 | 724.4 (1, 4126) | <.001 | 1 |
|  | Other clusters | 248.4 (78.63) | 42.33 | 238.2 (192.3-291.8) | 583.6 |  |  |  |
| **Selenium (ug/day)** |  |  |  |  |  |  |  |  |
|  | Cluster 1 | 40.08 (10.84) | 11.52 | 39.32 (32.93-46.55) | 90.7 | 1040.1 (1, 4126) | <.001 | 8 |
|  | Other clusters | 56.04 (17.12) | 14.41 | 53.75 (44.23-64.75) | 141.5 |  |  |  |
| **Submax cycle test borg score - stage 4** |  |  |  |  |  |  |  |  |
|  | Cluster 1 | 12.97 (3.471) | 6 | 13 (11-15) | 20 | 233.4 (1, 4126) | <.001 | 1 |
|  | Other clusters | 11.29 (3.328) | 6 | 12 (9-13) | 20 |  |  |  |
| **Estimated maximal power output (W) based on HR or RPE** |  |  |  |  |  |  |  |  |
|  | Cluster 1 | 127.7 (36.4) | 29.17 | 123.3 (103.7-148.8) | 278.3 | 1038.6 (1, 4126) | <.001 | 8 |
|  | Other clusters | 175 (49.25) | 29.17 | 170.2 (138.9-205.6) | 374.1 |  |  |  |
| **The age of the participant at the moment of measurement** |  |  |  |  |  |  |  |  |
|  | Cluster 1 | 65.8 (7.616) | 41 | 67 (61-71) | 81 | 371.4 (1, 4126) | <.001 | 2 |
|  | Other clusters | 60.65 (8.501) | 40 | 61 (54-67) | 82 |  |  |  |
| **Plasma glucose at t=120 min (mmol/l)** |  |  |  |  |  |  |  |  |
|  | Cluster 1 | 10.46 (5.001) | 2.3 | 8.8 (6.4-14.47) | 25.2 | 123.1 (1, 4126) | <.001 | 1 |
|  | Other clusters | 8.787 (4.398) | 2 | 7.1 (5.5-11.3) | 23.2 |  |  |  |
| **Diopter front mean left eye (Diopter)** |  |  |  |  |  |  |  |  |
|  | Cluster 1 | 44.31 (1.266) | 37.1 | 44.3 (43.4-45.1) | 49.5 | 1071.0 (1, 4126) | <.001 | 2 |
|  | Other clusters | 42.9 (1.344) | 34.7 | 42.9 (42.1-43.8) | 47.4 |  |  |  |
| **Radius back flat right eye (mm)** |  |  |  |  |  |  |  |  |
|  | Cluster 1 | 6.398 (0.2162) | 5.72 | 6.4 (6.26-6.54) | 7.2 | 849.9 (1, 4126) | <.001 | 1 |
|  | Other clusters | 6.62 (0.2429) | 5.75 | 6.61 (6.46-6.78) | 9.1 |  |  |  |
| **Radius back steep left eye (mm)** |  |  |  |  |  |  |  |  |
|  | Cluster 1 | 6.09 (0.2209) | 5.42 | 6.09 (5.94-6.24) | 6.78 | 871.7 (1, 4126) | <.001 | 1 |
|  | Other clusters | 6.315 (0.2403) | 5.58 | 6.31 (6.15-6.468) | 8.7 |  |  |  |
| **Radius back horizontal right eye (mm)** |  |  |  |  |  |  |  |  |
|  | Cluster 1 | 6.387 (0.2215) | 5.22 | 6.39 (6.25-6.53) | 7.13 | 837.2 (1, 4126) | <.001 | 1 |
|  | Other clusters | 6.616 (0.2541) | 5.75 | 6.61 (6.45-6.78) | 8.95 |  |  |  |
| **Radius back mean left eye (mm)** |  |  |  |  |  |  |  |  |
|  | Cluster 1 | 6.242 (0.2079) | 5.6 | 6.24 (6.11-6.38) | 6.89 | 941.4 (1, 4126) | <.001 | 4 |
|  | Other clusters | 6.464 (0.2306) | 5.73 | 6.46 (6.31-6.61) | 8.87 |  |  |  |
| **Diopter back mean left eye (Diopter)** |  |  |  |  |  |  |  |  |
|  | Cluster 1 | -6.417 (0.2156) | -7.1 | -6.4 (-6.5--6.3) | -5.8 | 963.2 (1, 4126) | <.001 | 1 |
|  | Other clusters | -6.195 (0.2213) | -7 | -6.2 (-6.3--6.1) | -4.5 |  |  |  |
| **Smallest Radius right eye (mm)** |  |  |  |  |  |  |  |  |
|  | Cluster 1 | 7.409 (0.2382) | 6.34 | 7.415 (7.263-7.57) | 8.17 | 1047.7 (1, 4126) | <.001 | 1 |
|  | Other clusters | 7.669 (0.2516) | 6.79 | 7.67 (7.51-7.83) | 8.7 |  |  |  |
| **Maximum K reading OS (Diopter)** |  |  |  |  |  |  |  |  |
|  | Cluster 1 | 45.64 (1.467) | 41.5 | 45.6 (44.6-46.5) | 53.2 | 1136.1 (1, 4126) | <.001 | 6 |
|  | Other clusters | 44.06 (1.428) | 37.9 | 44 (43.1-45) | 51.2 |  |  |  |
| **Visual acuity for the left eye** |  |  |  |  |  |  |  |  |
|  | Cluster 1 | 0.9266 (0.2504) | 0.07943 | 0.955 (0.7943-1.096) | 1.66 | 313.0 (1, 4126) | <.001 | 6 |
|  | Other clusters | 1.075 (0.2611) | 0.07943 | 1.096 (0.912-1.259) | 1.905 |  |  |  |
| **Visual acuity for the right eye** |  |  |  |  |  |  |  |  |
|  | Cluster 1 | 0.9121 (0.2505) | 0.07943 | 0.912 (0.7943-1.096) | 1.66 | 304.4 (1, 4126) | <.001 | 1 |
|  | Other clusters | 1.06 (0.2647) | 0.07943 | 1.096 (0.912-1.202) | 1.995 |  |  |  |
| **utility score SF6D (based on SF36)^d^** |  |  |  |  |  |  |  |  |
|  | Cluster 1 | 0.7546 (0.1149) | 0.374 | 0.753 (0.675-0.845) | 1 | 129.3 (1, 4126) | <.001 | 1 |
|  | Other clusters | 0.7955 (0.108) | 0.37 | 0.81 (0.74-0.881) | 1 |  |  |  |
| **SF36 physical functioning** |  |  |  |  |  |  |  |  |
|  | Cluster 1 | 72 (22.83) | 0 | 75 (55-90) | 100 | 297.9 (1, 4126) | <.001 | 1 |
|  | Other clusters | 83.35 (18.58) | 0 | 90 (75-95) | 100 |  |  |  |
| **How many siblings do you have (including deceased siblings)?** |  |  |  |  |  |  |  |  |
|  | Cluster 1 | 4.184 (3.493) | 0 | 3 (2-6) | 31 | 96.8 (1, 4126) | <.001 | 2 |
|  | Other clusters | 3.25 (2.549) | 0 | 3 (1-4) | 31 |  |  |  |
| **Number of persons in household within this income level** |  |  |  |  |  |  |  |  |
|  | Cluster 1 | 1.896 (0.6828) | 1 | 2 (2-2) | 6 | 205.2 (1, 4126) | <.001 | 1 |
|  | Other clusters | 2.315 (0.9949) | 1 | 2 (2-3) | 9 |  |  |  |

^a^Runs = Number of runs in which the variable occurs.

^b^Different versions of the GIT have been used, with differences in cutoff values. The differences have been cleaned and adjusted, into a new total score GIT_total [47].

^c^CST total time for null version 2 expressed in seconds. Convert minutes, seconds, and milliseconds into seconds [48].

^d^The SF-36 Health Survey is a standardized questionnaire used to assess health status and health-related quality of life [49].
