# Supplementary material for: Identification of Clusters in a Population With Obesity Using Machine Learning: Secondary Analysis of The Maastricht Study
Source: JMIR Med Inform. 2025 Feb 5;13:e64479. doi: 10.2196/64479 (PMC11840370; doi:10.2196/64479)
Supplement: Multimedia Appendix 6 [file medinform_v13i1e64479_app6.doc]

**Appendix 6.** Table with Cluster 1 (n=1458) compared to Clusters 2 and 3 combined (n=2670), categorical variables.

| **Variable** | Levels | Cluster 1 | Other clusters | Chi-square (*df*) | *P-*value | Runs^a^ |
| --- | --- | --- | --- | --- | --- | --- |
|  |  |  |  |  |  |  |
| **Groninger Intelligence Test (GIT) swol** |  |  |  |  |  |  |
|  | Correct, n(%)  Incorrect, n(%) | 600 (41.15)  858 (58.85) | 1470 (55.06)  1200 (44.94) | 72.4 (1) | <0.001 | 1 |
| **Blood pressure-lowering medication, ACE inhibitors (RAS inhibitors)** |  |  |  |  |  |  |
|  | No, n(%)  Yes, n(%) | 1161 (79.63)  297 (20.37) | 2320 (86.89)  350 (13.11) | 37.1 (1) | <0.001 | 3 |
| **Lipid-lowering medication, statins: HMG CoA reductase inhibitors** |  |  |  |  |  |  |
|  | No, n(%)  Yes, n(%) | 725 (49.73)  733 (50.27) | 1760 (65.92)  910 (34.08) | 102.5 (1) | <0.001 | 2 |
| **Ability to visit friends, neighbours or acquaintancees when you want to is:** |  |  |  |  |  |  |
|  | Bad, n(%)  Poor, n(%)  Fair, n(%)  Good, n(%)  Very good, n(%) | 10 (0.69)  45 (3.09)  139 (9.53)  618 (42.39)  646 (44.31) | 1 (0.04)  44 (1.65)  148 (5.54)  823 (30.82)  1654 (61.95) | 134.3 (4) | <0.001 | 1 |
| **Educational level categories** |  |  |  |  |  |  |
|  | High, n (%)  Low, n (%)  Medium, n (%) | 274 (18.79)  878 (60.22)  306 (20.99) | 930 (34.83)  918 (34.38)  822 (30.79) | 261.0 (2) | <0.001 | 1 |
| **Can you, fully independently, do 'heavy' household activities (e.g. mopping, cleaning the windows and vacuuming)? ^b^** |  |  |  |  |  |  |
|  | No, I need someone's help, n(%)  Yes, with great difficulty, n(%)  Yes, with some difficulty, n(%)  Yes, without any difficulty, n(%) | 127 (8.71)  137 (9.4)  343 (23.53)  851 (58.37) | 78 (2.92)  100 (3.75)  396 (14.83)  2096 (78.5) | 209.5 (3) | <0.001 | 1 |
| **Fast walking past 4 weeks^c^** |  |  |  |  |  |  |
|  | No, n(%)  Yes, n(%) | 935 (64.13)  523 (35.87) | 1142 (42.77)  1528 (57.23) | 171.2 (1) | <0.001 | 2 |
| **Social inadequacy: I prefer to stay on the background in a group of people** |  |  |  |  |  |  |
|  | ?, n(%)  Correct, n(%)  Incorrect, n(%) | 134 (9.19)  659 (45.2)  665 (45.61) | 184 (6.89)  931 (34.87)  1555 (58.24) | 60.6 (2) | <0.001 | 1 |
| **Employment status** |  |  |  |  |  |  |
|  | Employed, n(%)  Other, n(%)  Unemployed, n(%) | 313 (21.47)  34 (2.33)  1111 (76.2) | 1359 (50.9)  33 (1.24)  1278 (47.87) | 339.5 (2) | <0.001 | 1 |
| **Occupational category** |  |  |  |  |  |  |
|  | High occ class, n(%)  Intermediate occ class, n(%)  Low occ class, n(%)  Not working, n(%)  Other, n(%)  Self-employed, n(%) | 76 (5.21)  31 (2.13)  54 (3.7)  1236 (84.77)  4 (0.27)  57 (3.91) | 420 (15.73)  177 (6.63)  193 (7.23)  1486 (55.66)  5 (0.19)  389 (14.57) | 365.1 (5) | <0.001 | 4 |
| **Informal care received (last 6 months)** |  |  |  |  |  |  |
|  | No, n(%)  Yes, n(%) | 1254 (86.01)  204 (13.99) | 2466 (92.36)  204 (7.64) | 42.0 (1) | <0.001 | 1 |
| **Do you snore in all positions?** |  |  |  |  |  |  |
|  | I don't know, n(%)  No, n(%)  Yes, n(%) | 780 (53.5)  224 (15.36)  454 (31.14) | 1062 (39.78)  732 (27.42)  876 (32.81) | 99.8 (2) | <0.001 | 3 |

^a^Runs = Number of runs in which the variable occurs.

^b^The Groningen Activity Restriction Scale (GARS-4) is a non-disease-specific instrument to measure disability in activities of daily living (ADL) and instrumental activities of daily living (IADL) [50, 51].

^c^In a typical week during the past 4 weeks, did you walk fast [52]?
