# Supplementary material for: Identification of Clusters in a Population With Obesity Using Machine Learning: Secondary Analysis of The Maastricht Study
Source: JMIR Med Inform. 2025 Feb 5;13:e64479. doi: 10.2196/64479 (PMC11840370; doi:10.2196/64479)
Supplement: Multimedia Appendix 7 [file medinform_v13i1e64479_app7.doc]

**Appendix 7.** Table with Cluster 2 (n=1521) compared to Clusters 1 and 3 combined (n=2607), continuous variables.

| **Variable** | Cluster number | Mean (SD) | Minimum | Median (IQR) | Maximum | *F* test (*df*) | *P-*value | Runs^a^ |
| --- | --- | --- | --- | --- | --- | --- | --- | --- |
|  |  |  |  |  |  |  |  |  |
| **Mean of hourly mean diastolic blood pressure for night (23-8h); mmHg** |  |  |  |  |  |  |  |  |
|  | Cluster 2 | 68.51 (8.33) | 42.33 | 67.78 (62.94-73.28) | 103.8 | 205.6 (1, 4126) | <.001 | 1 |
|  | Other clusters | 64.95 (7.306) | 44.43 | 64.39 (60-69.44) | 104.3 |  |  |  |
| **Mean Arterial Pressure (MAP) based on day (8-23h) and night (23-8h); mmHg** |  |  |  |  |  |  |  |  |
|  | Cluster 2 | 91.31 (8.629) | 65 | 90.47 (85.5-96.16) | 124.6 | 237.0 (1, 4126) | <.001 | 6 |
|  | Other clusters | 87.35 (7.567) | 64.35 | 86.92 (82.29-91.81) | 125.5 |  |  |  |
| **Mean 7days diastolic blood pressure, all measurements (6.00-11.00h and 18.00-23.00h)** |  |  |  |  |  |  |  |  |
|  | Cluster 2 | 80.3 (8.622) | 59 | 80 (74-86) | 115 | 204.1 (1, 4126) | <.001 | 1 |
|  | Other clusters | 76.51 (7.966) | 47 | 76 (71-81) | 121 |  |  |  |
| **Peroneal nerve, conduction velocity, below fibular head to ankle (m/s)** |  |  |  |  |  |  |  |  |
|  | Cluster 2 | 44.61 (4.96) | 24.1 | 44.9 (41.6-48) | 59.8 | 187.1 (1, 4126) | <.001 | 1 |
|  | Other clusters | 46.69 (4.561) | 27 | 47 (43.9-49.6) | 65.4 |  |  |  |
| **Soup without legumes (g)** |  |  |  |  |  |  |  |  |
|  | Cluster 2 | 60.31 (71.46) | 0 | 35 (10-81.56) | 625 | 172.9 (1, 4126) | <.001 | 1 |
|  | Other clusters | 35.6 (48.91) | 0 | 20 (5-42) | 645 |  |  |  |
| **Milk semi-skimmed (g)** |  |  |  |  |  |  |  |  |
|  | Cluster 2 | 55.46 (107.1) | 0 | 1.2 (0-65.25) | 750 | 130.1 (1, 4126) | <.001 | 1 |
|  | Other clusters | 25.73 (60.4) | 0 | 0 (0-14) | 684 |  |  |  |
| **Energy intake (kcal/day)** |  |  |  |  |  |  |  |  |
|  | Cluster 2 | 2755 (506.2) | 1587 | 2659 (2391-3042) | 4178 | 5311.5 (1, 4126) | <.001 | 6 |
|  | Other clusters | 1749 (375) | 608 | 1755 (1491-2015) | 2937 |  |  |  |
| **Energy (KJ)** |  |  |  |  |  |  |  |  |
|  | Cluster 2 | 11563 (2144) | 6661 | 11149 (10009-12704) | 17525 | 5276.2 (1, 4126) | <.001 | 2 |
|  | Other clusters | 7333 (1573) | 2538 | 7366 (6269-8436) | 12310 |  |  |  |
| **C16:1 cis total (mg/day)** |  |  |  |  |  |  |  |  |
|  | Cluster 2 | 1297 (347.4) | 570.4 | 1235 (1071-1462) | 3104 | 3753.41 (1, 4126) | <.001 | 8 |
|  | Other clusters | 754.8 (221.1) | 111.7 | 743 (608-900.8) | 1644 |  |  |  |
| **ALA (g/day)** |  |  |  |  |  |  |  |  |
|  | Cluster 2 | 2123 (653.5) | 865.4 | 2018 (1678-2431) | 5839 | 2704.5 (1, 4126) | <.001 | 3 |
|  | Other clusters | 1266 (404.6) | 270.5 | 1235 (973.3-1518) | 3017 |  |  |  |
| **C18:1 n-6 cis (mg/day)** |  |  |  |  |  |  |  |  |
|  | Cluster 2 | 27.07 (24.72) | 0.8006 | 18.64 (10.63-34.71) | 180.1 | 728.4 (1, 4126) | <.001 | 2 |
|  | Other clusters | 11.91 (11.1) | 0.2943 | 8.369 (4.89-14.31) | 112 |  |  |  |
| **C18:1 n-10 trans (mg/day)** |  |  |  |  |  |  |  |  |
|  | Cluster 2 | 24.54 (22.15) | 0.1379 | 17.92 (11.09-29.52) | 193.1 | 735.2 (1, 4126) | <.001 | 2 |
|  | Other clusters | 10.83 (10.13) | 0.001766 | 8.635 (4.151-14.26) | 113.5 |  |  |  |
| **Pufa (g/day)** |  |  |  |  |  |  |  |  |
|  | Cluster 2 | 2.898 (1.019) | 0.9544 | 2.668 (2.204-3.366) | 7.178 | 2320.3 (1, 4126) | <.001 | 1 |
|  | Other clusters | 1.687 (0.5974) | 0.3865 | 1.63 (1.266-2.026) | 5.135 |  |  |  |
| **Retinol equivalents (ug/day)** |  |  |  |  |  |  |  |  |
|  | Cluster 2 | 1567 (851) | 355.2 | 1325 (1011-1831) | 5482 | 1371.1 (1, 4126) | <.001 | 8 |
|  | Other clusters | 836.8 (410.4) | 66.91 | 756.6 (572.3-1000) | 3610 |  |  |  |
| **Selenium (ug/day)** |  |  |  |  |  |  |  |  |
|  | Cluster 2 | 64.57 (16.38) | 32.79 | 61.58 (53.29-72.21) | 141.5 | 2807.2 (1, 4126) | <.001 | 4 |
|  | Other clusters | 42.14 (10.78) | 11.52 | 41.39 (35.11-48.97) | 102.2 |  |  |  |
| **Alpha-tocoferol (mg/day)** |  |  |  |  |  |  |  |  |
|  | Cluster 2 | 10.31 (3.248) | 4.251 | 9.684 (8.095-11.97) | 38.53 | 2435.8 (1, 4126) | <.001 | 4 |
|  | Other clusters | 6.321 (1.949) | 1.387 | 6.12 (4.986-7.45) | 16.42 |  |  |  |
| **alanine intake (g/day)** |  |  |  |  |  |  |  |  |
|  | Cluster 2 | 5.207 (1.456) | 2.166 | 4.856 (4.262-5.727) | 14.25 | 2728.5 (1, 4126) | <.001 | 4 |
|  | Other clusters | 3.343 (0.8369) | 0.9052 | 3.314 (2.806-3.839) | 10.42 |  |  |  |
| **Maximal Grip strength Overall (kg)** |  |  |  |  |  |  |  |  |
|  | Cluster 2 | 37.2 (10.92) | 7 | 37 (29-45) | 78 | 425.1 (1, 4126) | <.001 | 1 |
|  | Other clusters | 30.39 (9.806) | 3 | 28 (24-36) | 78 |  |  |  |
| **Maximal Grip strength dominant arm (kg)** |  |  |  |  |  |  |  |  |
|  | Cluster 2 | 36.22 (10.89) | 7 | 36 (28-44) | 72 | 412.2 (1, 4126) | <.001 | 4 |
|  | Other clusters | 29.52 (9.817) | 2 | 28 (23-36) | 72 |  |  |  |
| **Wrist circumference (cm)** |  |  |  |  |  |  |  |  |
|  | Cluster 2 | 18.1 (1.441) | 7 | 18 (17-19) | 24.5 | 300.2 (1, 4126) | <.001 | 2 |
|  | Other clusters | 17.31 (1.401) | 9.3 | 17 (16.2-18.2) | 30 |  |  |  |
| **Diopter front steep right eye (Diopter)** |  |  |  |  |  |  |  |  |
|  | Cluster 2 | 43.4 (1.477) | 35.7 | 43.4 (42.5-44.4) | 48.3 | 171.8 (1, 4126) | <.001 | 2 |
|  | Other clusters | 44.04 (1.547) | 37 | 44 (43.05-45) | 50.9 |  |  |  |
| **Radius front mean left eye (mm)** |  |  |  |  |  |  |  |  |
|  | Cluster 2 | 7.855 (0.2558) | 7.12 | 7.85 (7.68-8.02) | 9.38 | 168.9 (1, 4126) | <.001 | 2 |
|  | Other clusters | 7.746 (0.2631) | 6.82 | 7.74 (7.57-7.9) | 9.74 |  |  |  |
| **Diopter front mean right eye (Diopter)** |  |  |  |  |  |  |  |  |
|  | Cluster 2 | 42.98 (1.413) | 35.4 | 43 (42.1-43.9) | 47.3 | 169.2 (1, 4126) | <.001 | 2 |
|  | Other clusters | 43.59 (1.489) | 36.7 | 43.5 (42.7-44.6) | 49.4 |  |  |  |
| **Diopter front mean left eye (Diopter)** |  |  |  |  |  |  |  |  |
|  | Cluster 2 | 43.01 (1.4) | 36 | 43 (42.1-43.9) | 47.4 | 172.3 (1, 4126) | <.001 | 1 |
|  | Other clusters | 43.62 (1.475) | 34.7 | 43.6 (42.7-44.6) | 49.5 |  |  |  |
| **Smallest measured corneal thickness right eye (μm)** |  |  |  |  |  |  |  |  |
|  | Cluster 2 | 552.7 (31.58) | 436 | 554 (532-573) | 654 | 39.8 (1, 4126) | <.001 | 2 |
|  | Other clusters | 546.3 (31.2) | 440 | 546 (525-567.5) | 698 |  |  |  |
| **Maximum K reading OD (Diopter)** |  |  |  |  |  |  |  |  |
|  | Cluster 2 | 44.17 (1.557) | 38.8 | 44.1 (43.1-45.2) | 49.7 | 167.8 (1, 4126) | <.001 | 1 |
|  | Other clusters | 44.85 (1.661) | 39.2 | 44.8 (43.7-45.9) | 53.2 |  |  |  |

^a^Runs = Number of runs in which the variable occurs.
