# Supplementary material for: Identification of Clusters in a Population With Obesity Using Machine Learning: Secondary Analysis of The Maastricht Study
Source: JMIR Med Inform. 2025 Feb 5;13:e64479. doi: 10.2196/64479 (PMC11840370; doi:10.2196/64479)
Supplement: Multimedia Appendix 8 [file medinform_v13i1e64479_app8.doc]

**Appendix 8.** Table with Cluster 2 (n=1521) compared to Clusters 1 and 3 combined (n=2607), categorical variables.

| **Variable** | Levels | Cluster 2 | Other clusters | Chi-square (*df*) | *P-*value | Runs^a^ |
| --- | --- | --- | --- | --- | --- | --- |
|  |  |  |  |  |  |  |
| **Sex of the participant** |  |  |  |  |  |  |
|  | Female, n(%)  Male, n(%) | 524 (34.45)  997 (65.55) | 1722 (66.05)  885 (33.95) | 385.5 (1) | <0.001 | 1 |
| **Dozing while watching TV^b^** |  |  |  |  |  |  |
|  | High chance, n(%)  Moderate chance, n(%)  Slight chance, n(%)  Would never doze, n(%) | 267 (17.55)  571 (37.54)  473 (31.1)  210 (13.81) | 293 (11.24)  838 (32.14)  902 (34.6)  574 (22.02) | 74.1 (3) | <0.001 | 5 |

^a^Runs = Number of runs in which the variable occurs.

^b^How likely are you to doze off or fall asleep while watching TV [53]?
