# Supplementary material for: Identification of Clusters in a Population With Obesity Using Machine Learning: Secondary Analysis of The Maastricht Study
Source: JMIR Med Inform. 2025 Feb 5;13:e64479. doi: 10.2196/64479 (PMC11840370; doi:10.2196/64479)
Supplement: Multimedia Appendix 9 [file medinform_v13i1e64479_app9.doc]

**Appendix 9.** Table with Cluster 3 (n=1149) against Clusters 1 and 2 combined (n=2979), continuous variables.

| **Variable** | Cluster number | Mean (SD) | Minimum | Median (IQR) | Maximum | *F* test (*df*) | *P-*value | Runs^a^ |
| --- | --- | --- | --- | --- | --- | --- | --- | --- |
|  |  |  |  |  |  |  |  |  |
| **Relative systolic dipping between day (8-23h) and night (23-8h); %** |  |  |  |  |  |  |  |  |
|  | Cluster 3 | 10.09 (5.614) | -23.19 | 10.21 (6.47-13.86) | 31.68 | 54.3 (1, 4126) | <.001 | 1 |
|  | Other clusters | 8.549 (6.187) | -18.25 | 8.51 (4.4-12.61) | 30.24 |  |  |  |
| **Weighted standard deviation of systolic blood pressure during 24h** |  |  |  |  |  |  |  |  |
|  | Cluster 3 | 10.69 (2.82) | 5.098 | 10.25 (8.78-12.2) | 28.55 | 178.2 (1, 4126) | <.001 | 3 |
|  | Other clusters | 12.4 (3.97) | 5.112 | 11.57 (9.666-14.16) | 34.4 |  |  |  |
| **Weighted coefficient of variance of diastolic blood pressure during 24h** |  |  |  |  |  |  |  |  |
|  | Cluster 3 | 11 (3.363) | 4.949 | 10.27 (8.81-12.43) | 34.88 | 189.9 (1, 4126) | <.001 | 5 |
|  | Other clusters | 13.09 (4.708) | 4.944 | 12.02 (9.842-14.86) | 39.45 |  |  |  |
| **Mean 7days systolic blood pressure, morning (6.00-11.00h)** |  |  |  |  |  |  |  |  |
|  | Cluster 3 | 126 (13.5) | 92 | 125 (117-134) | 192 | 181.1 (1, 4126) | <.001 | 1 |
|  | Other clusters | 132.9 (15.39) | 87 | 132 (123-141) | 198 |  |  |  |
| **MMSE total score^b^** |  |  |  |  |  |  |  |  |
|  | Cluster 3 | 29.27 (0.9099) | 25 | 30 (29-30) | 30 | 187 (1, 4126) | <.001 | 2 |
|  | Other clusters | 28.62 (1.508) | 18 | 29 (28-30) | 30 |  |  |  |
| **Concept Shifting Test (CST) total time for trail C (s)^c^** |  |  |  |  |  |  |  |  |
|  | Cluster 3 | 30.01 (8.852) | 12.91 | 28.34 (23.72-34.88) | 72.38 | 356.5 (1, 4126) | <.001 | 2 |
|  | Other clusters | 40.05 (17.17) | 14.41 | 36.19 (28.94-46.59) | 176 |  |  |  |
| **Concept Shifting Test (CST) 2 (s)^c^** |  |  |  |  |  |  |  |  |
|  | Cluster 3 | 4.379 (0.9288) | 2 | 4 (4-5) | 8 | 342.7 (1, 4126) | <.001 | 2 |
|  | Other clusters | 5.233 (1.454) | 2 | 5 (4-6) | 13 |  |  |  |
| **Concept Shifting Test (CST) total time for null version 2 (s)^c^** |  |  |  |  |  |  |  |  |
|  | Cluster 3 | 4.865 (0.8794) | 2.44 | 4.75 (4.25-5.35) | 8.63 | 349.7 (1, 4126) | <.001 | 5 |
|  | Other clusters | 5.704 (1.419) | 2.97 | 5.44 (4.72-6.35) | 13.81 |  |  |  |
| **Information processing speed (cognitive domain)** |  |  |  |  |  |  |  |  |
|  | Cluster 3 | 0.2835 (0.6544) | -1.781 | 0.2907 (-0.1596-0.733) | 2.328 | 520.9 (1, 4126) | <.001 | 1 |
|  | Other clusters | -0.3395 (0.8312) | -3.556 | -0.2872 (-0.8728-0.2334) | 2.028 |  |  |  |
| **Overall cognitive functioning** |  |  |  |  |  |  |  |  |
|  | Cluster 3 | 0.2349 (0.5702) | -1.669 | 0.2568 (-0.1481-0.6492) | 1.719 | 526.2 (1, 4126) | <.001 | 5 |
|  | Other clusters | -0.3088 (0.7212) | -2.621 | -0.2697 (-0.7772-0.2044) | 2.683 |  |  |  |
| **Daily intake of herring, sardines/pilchards (g), based on a food frequency questionnaire.** |  |  |  |  |  |  |  |  |
|  | Cluster 3 | 1.478 (2.469) | 0 | 0.1 (0-2.059) | 15.26 | 57.6 (1, 4126) | <.001 | 1 |
|  | Other clusters | 2.603 (4.784) | 0 | 0.9091 (0-3.111) | 43 |  |  |  |
| **C16:0 (mg/day)** |  |  |  |  |  |  |  |  |
|  | Cluster 3 | 11672 (2947) | 4484 | 11601 (9482-13696) | 21248 | 394.3 (1, 4126) | <.001 | 8 |
|  | Other clusters | 15681 (6593) | 2786 | 15121 (10640-19520) | 48216 |  |  |  |
| **Retinol equivalents (ug/day)** |  |  |  |  |  |  |  |  |
|  | Cluster 3 | 862.2 (390.5) | 99.78 | 790.4 (596.1-1032) | 3138 | 199 (1, 4126) | <.001 | 1 |
|  | Other clusters | 1200 (773.6) | 66.91 | 997.5 (704.7-1468) | 5482 |  |  |  |
| **Vitamin C (mg/day)** |  |  |  |  |  |  |  |  |
|  | Cluster 3 | 110.8 (51.23) | 21.93 | 102.2 (74.12-135) | 359.6 | 116.3 (1, 4126) | <.001 | 3 |
|  | Other clusters | 136.1 (73.02) | 13.37 | 121.3 (85.8-168.3) | 556.3 |  |  |  |
| **Folates (ug/day). Nutrient intake is calculated by multiplying food intake by the food composition table (NEVO) 2011** |  |  |  |  |  |  |  |  |
|  | Cluster 3 | 231.8 (57.89) | 78.39 | 226 (190.4-267) | 485.7 | 221.1 (1, 4126) | <.001 | 1 |
|  | Other clusters | 276.1 (94.34) | 63.44 | 265.3 (211.2-326.2) | 718.7 |  |  |  |
| **Folates equivalents (ug/day). Nutrient intake is calculated by multiplying food intake by the food composition table (NEVO) 2011** |  |  |  |  |  |  |  |  |
|  | Cluster 3 | 249.6 (71.09) | 82.79 | 240 (199.6-293.4) | 655.3 | 231.9 (1, 4126) | <.001 | 3 |
|  | Other clusters | 307.3 (120.7) | 66.78 | 283.9 (224-366.7) | 844.4 |  |  |  |
| **Sodium intake (mg/day)** |  |  |  |  |  |  |  |  |
|  | Cluster 3 | 7.439 (2.308) | 0 | 7.83 (5.965-9.573) | 10 | 376.5 (1, 4126) | <.001 | 6 |
|  | Other clusters | 5.196 (3.646) | 0 | 5.352 (1.772-8.76) | 10 |  |  |  |
| **Increase in heart rate during submaximal cycle test** |  |  |  |  |  |  |  |  |
|  | Cluster 3 | 46.81 (13.18) | 0 | 48 (38-56) | 90 | 367.6 (1, 4126) | <.001 | 4 |
|  | Other clusters | 37.05 (15.19) | 0 | 37 (27-48) | 102 |  |  |  |
| **Mean retinal sensitivity OD (right eye); dB** |  |  |  |  |  |  |  |  |
|  | Cluster 3 | 28.14 (1.457) | 10.52 | 28.33 (27.56-29) | 30.89 | 163.3 (1, 4126) | <.001 | 1 |
|  | Other clusters | 26.86 (3.271) | 0 | 27.52 (26.33-28.46) | 31.46 |  |  |  |
| **Retinal sensitivity at coordinate 6 for the right eye; dB** |  |  |  |  |  |  |  |  |
|  | Cluster 3 | 27.64 (1.831) | 4.9 | 27.7 (26.7-28.8) | 31.3 | 178.5 (1, 4126) | <.001 | 4 |
|  | Other clusters | 25.97 (4.072) | 0 | 26.8 (25.4-28) | 31.7 |  |  |  |
| **Retinal sensitivity at coordinate 9 for the right eye; dB** |  |  |  |  |  |  |  |  |
|  | Cluster 3 | 28.81 (1.693) | 13 | 29 (28.1-29.8) | 31.9 | 138.3 (1, 4126) | <.001 | 1 |
|  | Other clusters | 27.33 (4.12) | 0 | 28.2 (26.9-29.2) | 32.9 |  |  |  |
| **Retinal sensitivity at coordinate 6 for the left eye; dB** |  |  |  |  |  |  |  |  |
|  | Cluster 3 | 27.42 (1.793) | 0 | 27.5 (26.5-28.6) | 32.5 | 144.8 (1, 4126) | <.001 | 2 |
|  | Other clusters | 26.09 (3.577) | 0 | 26.7 (25.4-27.9) | 32.5 |  |  |  |
| **HbA1c (%)** |  |  |  |  |  |  |  |  |
|  | Cluster 3 | 5.661 (0.7241) | 4.346 | 5.535 (5.261-5.902) | 9.836 | 169.5 (1, 4126) | <.001 | 1 |
|  | Other clusters | 6.092 (1.026) | 3.431 | 5.81 (5.444-6.45) | 14.14 |  |  |  |
| **Pulse pressure (mmHg)** |  |  |  |  |  |  |  |  |
|  | Cluster 3 | 54.61 (11.55) | 26 | 53 (47-61) | 112 | 192.3 (1, 4126) | <.001 | 4 |
|  | Other clusters | 61.4 (14.95) | 26 | 59 (51-70) | 156 |  |  |  |
| **Diopter front mean left eye (Diopter)** |  |  |  |  |  |  |  |  |
|  | Cluster 3 | 42.76 (1.252) | 34.7 | 42.8 (42-43.6) | 46.7 | 320.8 (1, 4126) | <.001 | 1 |
|  | Other clusters | 43.64 (1.484) | 36 | 43.6 (42.7-44.6) | 49.5 |  |  |  |
| **Minimum radius of curvature for both the anterior and posterior corneal surface right eye (mm)** |  |  |  |  |  |  |  |  |
|  | Cluster 3 | 7.699 (0.225) | 6.95 | 7.7 (7.55-7.84) | 8.6 | 328.4 (1, 4126) | <.001 | 2 |
|  | Other clusters | 7.533 (0.2765) | 6.34 | 7.52 (7.35-7.71) | 8.7 |  |  |  |
| **Radius back horizontal right eye (mm)** |  |  |  |  |  |  |  |  |
|  | Cluster 3 | 6.644 (0.2318) | 5.89 | 6.63 (6.5-6.8) | 8.09 | 283.8 (1, 4126) | <.001 | 1 |
|  | Other clusters | 6.493 (0.2672) | 5.22 | 6.49 (6.32-6.66) | 8.95 |  |  |  |
| **Diopter back mean left eye (Diopter)** |  |  |  |  |  |  |  |  |
|  | Cluster 3 | -6.166 (0.2097) | -6.9 | -6.2 (-6.3--6) | -4.5 | 333 (1, 4126) | <.001 | 7 |
|  | Other clusters | -6.315 (0.2431) | -7.1 | -6.3 (-6.5--6.2) | -5.2 |  |  |  |
| **Smallest Radius right eye (mm)** |  |  |  |  |  |  |  |  |
|  | Cluster 3 | 7.698 (0.2244) | 6.95 | 7.69 (7.55-7.84) | 8.6 | 326.3 (1, 4126) | <.001 | 1 |
|  | Other clusters | 7.531 (0.2807) | 6.34 | 7.52 (7.35-7.71) | 8.7 |  |  |  |
| **Maximum K reading OS (Diopter)** |  |  |  |  |  |  |  |  |
|  | Cluster 3 | 43.9 (1.271) | 39 | 43.9 (43.1-44.7) | 48.2 | 339.7 (1, 4126) | <.001 | 3 |
|  | Other clusters | 44.9 (1.665) | 37.9 | 44.9 (43.8-45.9) | 53.2 |  |  |  |
| **Carotid to femoral pulse wave velocity (m/s)** |  |  |  |  |  |  |  |  |
|  | Cluster 3 | 8.57 (1.746) | 4.96 | 8.24 (7.36-9.44) | 17.84 | 173.9 (1, 4126) | <.001 | 1 |
|  | Other clusters | 9.578 (2.354) | 3.52 | 9.12 (7.92-10.72) | 24.8 |  |  |  |
| **LogMar visualy acuity (Logarithm of the Minimum Angle of Resolution) for the left eye** |  |  |  |  |  |  |  |  |
|  | Cluster 3 | -0.03762 (0.1141) | -0.26 | -0.06 (-0.1-0.02) | 1.1 | 177.8 (1, 4126) | <.001 | 1 |
|  | Other clusters | 0.03135 (0.1603) | -0.28 | 0 (-0.06-0.08) | 1.1 |  |  |  |
| **SF36 physical functioning^d^** |  |  |  |  |  |  |  |  |
|  | Cluster 3 | 88.54 (13.86) | 10 | 95 (85-100) | 100 | 333.6 (1, 4126) | <.001 | 8 |
|  | Other clusters | 75.79 (22.04) | 0 | 80 (65-95) | 100 |  |  |  |
| **SF36 general health^d^** |  |  |  |  |  |  |  |  |
|  | Cluster 3 | 70.76 (15.32) | 10 | 70 (60-80) | 100 | 240.7 (1, 4126) | <.001 | 2 |
|  | Other clusters | 61.27 (18.42) | 0 | 65 (50-75) | 100 |  |  |  |
| **Summary score general self-efficacy^e^** |  |  |  |  |  |  |  |  |
|  | Cluster 3 | 62.12 (7.289) | 31 | 62 (58-66) | 80 | 190.8 (1, 4126) | <.001 | 2 |
|  | Other clusters | 58.32 (8.159) | 26 | 59 (53-63) | 80 |  |  |  |
| **Summary score subscale effort general self-efficacy^e^** |  |  |  |  |  |  |  |  |
|  | Cluster 3 | 23.67 (3.383) | 6 | 24 (22-26) | 30 | 164.4 (1, 4126) | <.001 | 1 |
|  | Other clusters | 21.93 (4.101) | 6 | 22 (19-24) | 30 |  |  |  |
| **Aggression (mean score)^f^** |  |  |  |  |  |  |  |  |
|  | Cluster 3 | 2.049 (0.5143) | 1 | 2 (1.65-2.35) | 5 | 152.6 (1, 4126) | <.001 | 1 |
|  | Other clusters | 2.299 (0.6052) | 1 | 2.25 (1.85-2.7) | 4.65 |  |  |  |
| **Aggression (sum score)^f^** |  |  |  |  |  |  |  |  |
|  | Cluster 3 | 40.86 (10.35) | 20 | 40 (33-47) | 100 | 153.9 (1, 4126) | <.001 | 1 |
|  | Other clusters | 45.92 (12.23) | 20 | 45 (37-54) | 93 |  |  |  |
| **Hostility (subscale sum score)^f^** |  |  |  |  |  |  |  |  |
|  | Cluster 3 | 15.24 (5.158) | 8 | 15 (11-18) | 40 | 179.3 (1, 4126) | <.001 | 4 |
|  | Other clusters | 17.94 (6.024) | 8 | 18 (14-22) | 40 |  |  |  |

^a^Runs = Number of runs in which the variable occurs.

^b^Mini-Mental State Examination, cognition, neurological [54].

^c^CST total time for null version 2 expressed in seconds. Convert minutes, seconds, and milliseconds into seconds [48].

^d^The SF-36 Health Survey is a standardized questionnaire used to assess health status and health-related quality of life [55, 56].

^e^Perceived self-efficacy is defined as the belief of a person in his or her ability to organize and execute certain behaviours that are necessary in order to produce given attainments [57].

^f^The adult Aggression Questionnaire is a measure of aggression with 3 subscales: hostility, anger, and verbal aggression [58, 59].
