# Supplementary material for: Identification of Clusters in a Population With Obesity Using Machine Learning: Secondary Analysis of The Maastricht Study
Source: JMIR Med Inform. 2025 Feb 5;13:e64479. doi: 10.2196/64479 (PMC11840370; doi:10.2196/64479)
Supplement: Multimedia Appendix 10 [file medinform_v13i1e64479_app10.doc]

**Appendix 10.** Table with Cluster 3 (n=1149) against Clusters 1 and 2 combined (n=2979), categorical variables.

| **Variable** | Levels | Cluster 3 | Other clusters | Chi-square (*df*) | *P-*value | Runs^a^ |
| --- | --- | --- | --- | --- | --- | --- |
|  |  |  |  |  |  |  |
| **Groninger Intelligence Test (GIT) Total** |  |  |  |  |  |  |
|  | Correct, n(%)  Incorrect, n(%) | 1097 (95.47)  52 (4.53) | 2417 (81.13)  562 (18.87) | 133.5 (1) | <0.001 | 1 |
| **Educational level 3 categories** |  |  |  |  |  |  |
|  | High (higher professional education/ university education), n (%)  Low (no education/ primary education/ lower vocational education), n (%)  Middle (intermediate vocational education/ higher secondary education/ higher vocational education), n (%) | 507 (44.13)  84 (7.31)  558 (48.56) | 828 (27.79)  831 (27.9)  1320 (44.31) | 230.2 (2) | <0.001 | 2 |
| **Educational level 3 categories (N_Education_3cat)** |  |  |  |  |  |  |
|  | High (Higher vocational education or university education), n (%)  Low (No education, (un)completed primary education, or lower vocational education), n (%)  Medium (Intermediate vocational education or higher secondary education), n (%) | 475 (41.34)  281 (24.46)  393 (34.2) | 729 (24.47)  1515 (50.86)  735 (24.67) | 241.3 (2) | <0.001 | 2 |
| **Feeling nervous^b^** |  |  |  |  |  |  |
|  | A good bit of the time, n(%)  A little bit of the time, n(%)  All of the time, n(%)  Most of the time, n(%)  None of the time, n(%)  Some of the time, n(%) | 20 (1.74)  570 (49.61)  0 (0)  5 (0.44)  332 (28.89)  222 (19.32) | 132 (4.43)  1165 (39.11)  26 (0.87)  58 (1.95)  647 (21.72)  951 (31.92) | 124.8 (5) | <0.001 | 4 |
| **Do you doze off during the day?** |  |  |  |  |  |  |
|  | No, n(%)  Yes, n(%) | 827 (71.98)  322 (28.02) | 1475 (49.51)  1504 (50.49) | 168.7 (1) | <0.001 | 1 |
| **My friends find me quite quarrelsome^c^** |  |  |  |  |  |  |
|  | Agree, n(%)  Completely agree, n(%)  Completely disagree, n(%)  Disagree, n(%)  Undecided, n(%) | 13 (1.13)  12 (1.04)  825 (71.8)  251 (21.85)  48 (4.18) | 61 (2.05)  74 (2.48)  1893 (63.54)  622 (20.88)  329 (11.04) | 63.9 (4) | <0.001 | 2 |

^a^Runs = Number of runs in which the variable occurs.

^b^The SF-36 Health Survey is a standardized questionnaire used to assess health status and health-related quality of life [55, 56].

^c^The adult Aggression Questionnaire is a measure of aggression with 3 subscales: hostility, anger, and verbal aggression [58, 59].
